# Evolving the naturally compromised chorismate mutase from *Mycobacterium tuberculosis* to top performance

**DOI:** 10.1074/jbc.RA120.014924

**Published:** 2020-10-09

**Authors:** Jūrate˙ Fahrig-Kamarauskait≑, Kathrin Würth-Roderer, Helen V. Thorbjørnsrud, Susanne Mailand, Ute Krengel, Peter Kast

**Affiliations:** 1 Laboratory of Organic Chemistry, ETH Zurich, Zurich, Switzerland; 2 Department of Chemistry, University of Oslo, Oslo, Norway

**Keywords:** catalytic efficiency, conformational change, crystal structure, directed evolution, enzyme catalysis, enzyme mutation, molecular evolution, protein structure, structure-activity relationship, X-ray crystallography

## Abstract

Chorismate mutase (CM), an essential enzyme at the branch-point of the shikimate pathway, is required for the biosynthesis of phenylalanine and tyrosine in bacteria, archaea, plants, and fungi. MtCM, the CM from *Mycobacterium tuberculosis*, has less than 1% of the catalytic efficiency of a typical natural CM and requires complex formation with 3-deoxy-d-*arabino*-heptulosonate 7-phosphate synthase for high activity. To explore the full potential of MtCM for catalyzing its native reaction, we applied diverse iterative cycles of mutagenesis and selection, thereby raising *k*_cat_/*K_m_* 270-fold to 5 × 10^5^ m^−1^s^−1^, which is even higher than for the complex. Moreover, the evolutionarily optimized autonomous MtCM, which had 11 of its 90 amino acids exchanged, was stabilized compared with its progenitor, as indicated by a 9 °C increase in melting temperature. The 1.5 Å crystal structure of the top-evolved MtCM variant reveals the molecular underpinnings of this activity boost. Some acquired residues (*e.g.* Pro^52^ and Asp^55^) are conserved in naturally efficient CMs, but most of them lie beyond the active site. Our evolutionary trajectories reached a plateau at the level of the best natural enzymes, suggesting that we have exhausted the potential of MtCM. Taken together, these findings show that the scaffold of MtCM, which naturally evolved for mediocrity to enable inter-enzyme allosteric regulation of the shikimate pathway, is inherently capable of high activity.

The potential of a microbial cell to respond to changing environmental conditions is reflected in its ability to reorganize the metabolic flux. Allosteric feedback regulation of enzymes, where effector binding to an allosteric site tunes the activity of a distant active site, instantaneously achieves such a response. Typically, this type of regulation employs allosteric sites on the enzyme itself. Alternatively, an allosteric site can be temporarily provided by transient protein-protein interactions, also called “inter-enzyme allostery”, as first described for shikimate pathway enzymes from *Mycobacterium tuberculosis* H37Rv (1) and more recently also from *Corynebacterium glutamicum* (2).

Indeed, tight feedback regulation of metabolic flux is particularly important for the shikimate pathway, because it metabolizes a significant portion of organic carbon for the biosynthesis of energetically costly aromatic compounds in bacteria, archaea, fungi, algae, plants, and protozoan parasites (3–5). The pathway starts with the condensation of phosphoenolpyruvate and erythrose-4-phosphate to form 3-deoxy-d-*arabino*-heptulosonate 7-phosphate (DAHP) catalyzed by DAHP synthase (DS). Six subsequent enzymatic steps lead to the biosynthesis of the branch-point metabolite chorismate, the last common precursor of the aromatic amino acids and other essential aromatic compounds. The first committed step toward l-phenylalanine and l-tyrosine is the conversion of chorismate to prephenate (Fig. 1*A*), catalyzed by chorismate mutase (CM). Because DS and CM are the central nodes of the shikimate pathway, organisms have developed a variety of strategies both at the genetic (4) and protein (6–10) level to regulate these enzymes. In *M. tuberculosis*, CM (MtCM, a dimer encoded by *Rv0948c*, *aroQ_δ_*) has evolved to transiently interact with DS (MtDS, a tetramer encoded by *Rv2178c*, *aroG*) to form a heterooctameric complex (Fig. 1*B*). Only the complexed, but not the free dimeric MtCM, is responsive to feedback regulation by Phe and Tyr (1, 11).

**Figure 1.**
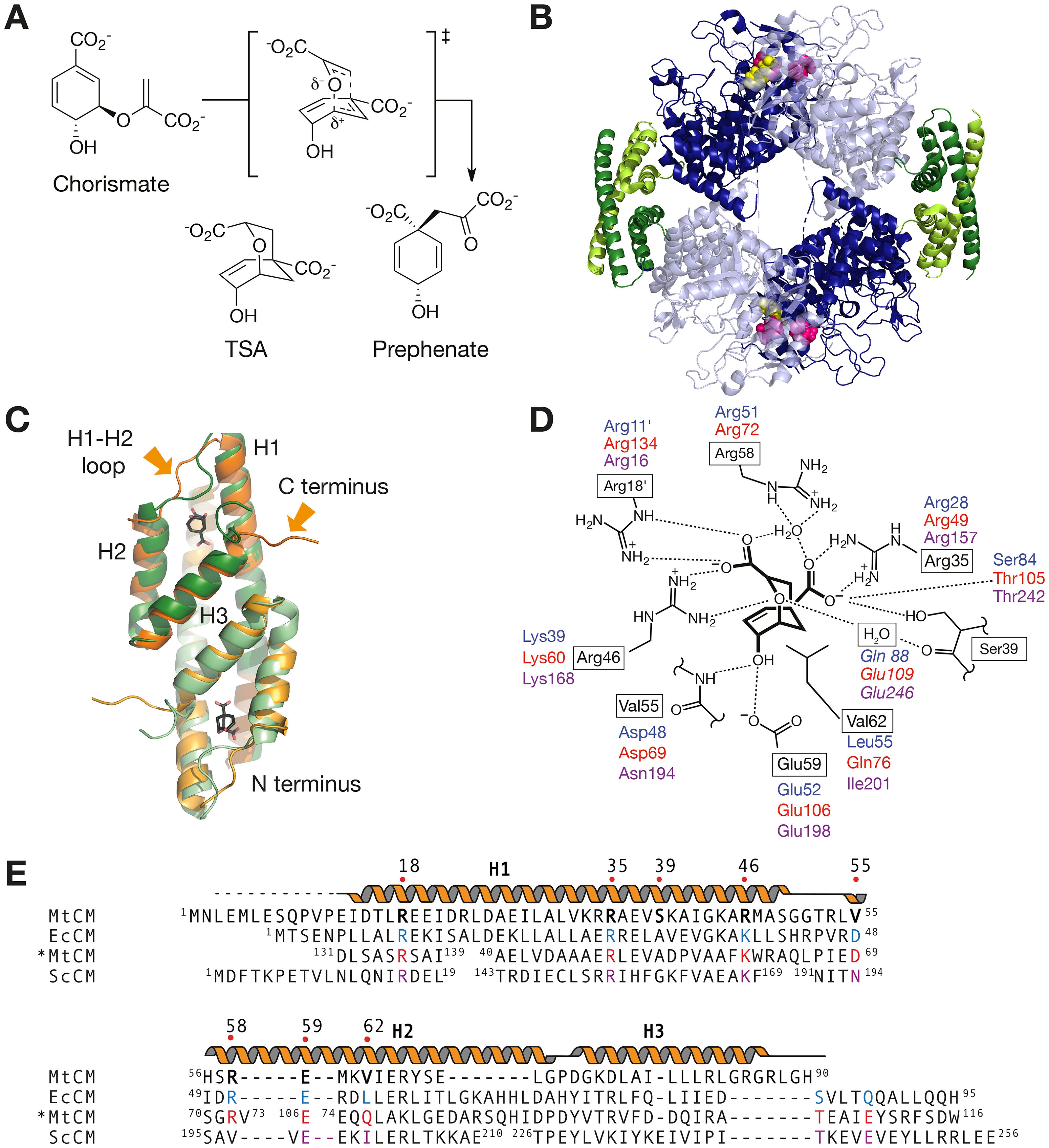
**Biosynthetic reaction, structure, active site, and sequences of AroQ chorismate mutases.** *A*, CM accelerates the Claisen rearrangement of chorismate to prephenate. The reaction proceeds via a transition state with chair-like geometry. Also shown is Bartlett's *endo*-oxa-bicyclic transition state analog (*TSA*, (74)), which is a good inhibitor of CMs. *B*, crystal structure of the MtCM-MtDS complex with the bound feedback inhibitors Phe (*pink spheres*) and Tyr (*yellow spheres*) (PDB ID: 5CKX). Two dimers of MtCM (subunits shown in *two shades of green*) bind to an MtDS homotetramer (shown in *two shades of blue*). *C*, structural superimposition of the poorly active malate-bound MtCM (*two shades of orange*, malate not shown; PDB ID: 2VKL) and MtDS-bound activated MtCM (*two shades of green*, TSA in *black*; PDB ID: 2W1A). Helices H1, H2, and H3 of one subunit are labeled; regions showing different conformations (H1-H2 loop and C terminus) are indicated by *arrows*. *D*, scheme of active site residues (*boxed*, *black*) of MtCM bound to TSA in its naturally activated state in complex with MtDS (PDB ID: 2W1A). Analogous prominent catalytic site residues in EcCM (*blue*; *labels in first line*), *MtCM (*red*; *second line*), and ScCM (*magenta*; *third line*) are shown together with their polar interactions (*dotted lines*). Italicized residues have no correspondence in MtCM. Residues projecting from a different protomer are labeled with a prime (′). *E*, sequence alignment of MtCM with the CM subclass prototypes AroQ_α_ (*EcCM*), AroQ_β_ (*ScCM*), and AroQ_γ_ (*MtCM) with an emphasis on matching analogous active site residues as in (*D*). *Helical*, *straight*, and *dotted lines* above the MtCM sequence indicate α-helices, loops, and unresolved parts of the MtCM structure in the MtCM-MtDS complex (PDB ID: 2W1A), respectively.

MtCM represents the structurally simple AroQ_δ_ subclass of CMs composed of two intertwined three-helix subunits (Fig. 1*C*) (1, 11). Other prototypical fold variants within the structurally and evolutionarily related α-helical AroQ class of CMs (12) comprise the CM domain of the bifunctional CM-prephenate dehydratase from *Escherichia coli* (EcCM, subclass AroQ_α_ (13)), the elaborate eukaryotic 12-helical CM from *Saccharomyces cerevisiae* (ScCM, AroQ_β_ (14, 15)), and the secreted enzyme from *M. tuberculosis* (*MtCM, AroQ_γ_ (16)). In stark contrast to the α, β, and γ CM subclasses, MtCM utilizes an arginine residue (Arg^46^) instead of an otherwise absolutely conserved lysine to promote the electrostatic catalysis (17) of the Claisen rearrangement of chorismate (Fig. 1, *D* and *E*) (11). Furthermore, MtCM is shorter at the C terminus and lacks the active site residue homologous to Gln^88^ in EcCM (18, 19), Glu^109^ in *MtCM (20), and Glu^246^ in ScCM (21). Despite these dramatic deviations from the consensus active site, MtCM is capable of catalyzing the conversion of chorismate to prephenate with a high catalytic efficiency (*k*_cat_/*K_m_* = 2.4 × 10^5^ m^−1^ s^−1^) (11). However, this can only be achieved in the presence of MtDS. On its own, MtCM is a mediocre enzyme catalyzing the reaction by two orders of magnitude less efficiently (*k*_cat_/*K_m_* of 1.8 × 10^3^ m^−1^ s^−1^) than the prototypical CMs of the other subclasses (16, 18, 21). In fact, the poor activity of the MtCM dimer is essential for effective shikimate pathway regulation, exerted through inter-enzyme allostery in *M. tuberculosis*. We (1, 11) and others (22, 23) have shown that binding of the allosteric feedback inhibitors Phe and Tyr to MtDS induces MtCM-MtDS complex dissociation and thereby a shift from high to low intracellular CM activity, providing tight control over cytoplasmic aromatic amino acid concentrations.

In this study, we probed the structural and mechanistic requirements for the activity switch of MtCM and whether or not interaction with MtDS is mandatory for efficient catalysis. We have employed diverse cycles of directed evolution to improve the mediocre efficiency of MtCM as the prototype for catalytically impaired AroQ_δ_ enzymes (1, 2, 11). Our results reveal mutation patterns and structural changes responsible for high activity and demonstrate that MtCM is inherently capable of efficient catalysis despite the lack of crucial active site residues, which are otherwise strongly conserved in the AroQ family. Thus, retaining such catalytic residues is not a strict requirement for achieving maximum catalytic prowess. More important is the proper positioning and orientation of analogous functional groups.

## Results

### Evolutionary strategy using in vivo selection

**Figure 2.**
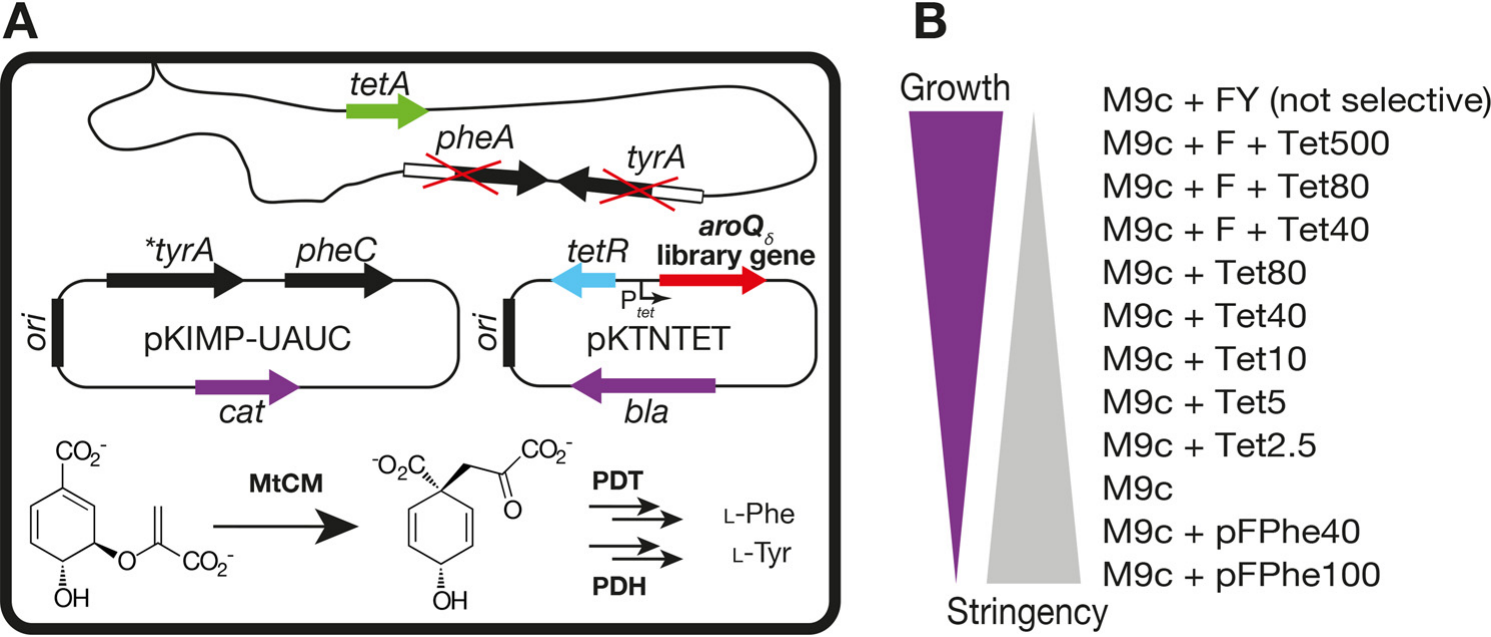
***In vivo* selection system for directed evolution.** *A*, CM selection system based on *E. coli* KA12/pKIMP-UAUC. The strain KA12 has a deletion of the CM-encoding bifunctional *pheA* and *tyrA* genes. This defect is only partially complemented by monofunctional versions of prephenate dehydratase (*PDT*, encoded by *pheC*) and prephenate dehydrogenase (*PDH*, encoded by *tyrA) on plasmid pKIMP-UAUC with the replication origin *ori*_p15A_. Consequently, survival on minimal medium lacking Phe (*F*) and Tyr (*Y*) requires introduction of a functional CM library gene (*aroQ_δ_*) on the compatible pKTNTET-based plasmid (*ori*_pUC_). The genes *bla*, *cat*, *pheA*, *tyrA*, *tetA*, and *tetR* encode β-lactamase, chloramphenicol acetyltransferase, CM-PDT, CM-PDH, a tetracycline efflux pump, and the repressor of the *tetA* promoter (P*_tet_*), respectively. *B*, selection stringencies used for MtCM evolution. The minimal medium M9c was provided with Phe, Tyr, tetracycline (*Tet*, *aroQ_δ_* inducer; concentration in ng/ml), and dl*-para*-fluoro-phenylalanine (*pFPhe*; concentration in μm) as indicated above. pFPhe causes cell death if incorporated to a significant extent into cellular proteins instead of Phe (75). This is because of the inability of the cell's phenylalanyl-tRNA synthetase to distinguish between the natural amino acid and its analog pFPhe, resulting in accumulation of faulty proteins (76, 77). By adding pFPhe to the medium, we exploit and adapt this observation for a new selection strategy favoring highly efficient CM variants, which provide sufficient endogenously produced Phe to outcompete the toxic pFPhe.

**Figure 3.**
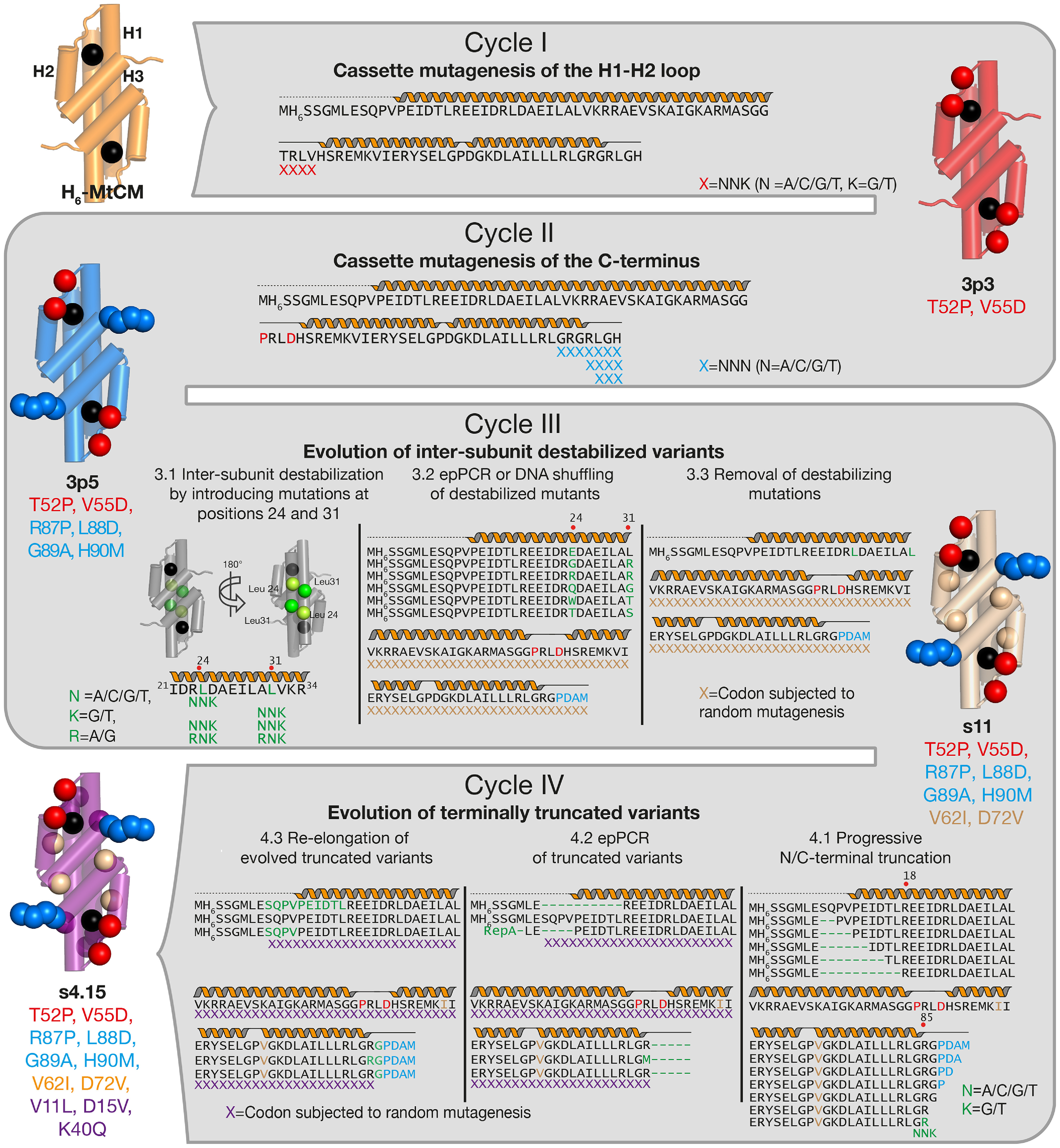
**Directed evolution strategy.** Two cycles of cassette mutagenesis (cycle I, H1-H2 loop; cycle II, C terminus) were followed by two cycles of perturbation-compensation (*green letters*) involving epPCR and DNA shuffling (cycle III, inter-subunit destabilization; cycle IV, progressive terminal truncations). The four evolutionary cycles and mutated residues are shown in different colors (I, *red*; II, *light blue*; III, *orange*; IV, *purple*). Representative variant names are listed. The mutated positions are indicated as *spheres* mapped onto CM *cartoons*; the *black sphere* pinpoints the location of the active site.

To explore the intrinsic potential of MtCM for efficient catalysis, we enlisted the strategy of directed evolution, a powerful experimental tool for probing and improving key features of enzymes (24–29). We applied several cycles of mutagenesis and selection to identify mutations in MtCM that increase CM efficiency in the absence of interacting MtDS (Fig. 2 and Fig. 3). Because CMs are essential metabolic enzymes, our evolutionary approach could take advantage of direct selection using the previously described *E. coli* CM knockout strain KA12/pKIMP-UAUC (30) (Fig. 2*A*). Transformation with plasmid libraries carrying mutagenized *M. tuberculosis aroQ_δ_* genes allows for rapid identification of survivors and thereby of active enzymes from typically 10^6^ variants per evolutionary round under the appropriate selective conditions (Fig. 2*B*).

As the starting point for the directed evolution we constructed H_6_-MtCM, an N-terminally His-tagged version of the WT MtCM that facilitates biochemical characterization. The catalytic efficiency (*k*_cat_/*K_m_* = 9 × 10^2^ m^−1^ s^−1^) of H_6_-MtCM is preserved within a factor of two of the parental enzyme (Table 1). H_6_-MtCM does not support the growth of KA12/pKIMP-UAUC on minimal medium lacking Tyr and Phe when its gene is introduced on the high-copy plasmid pKTNTET, downstream of the tightly repressed P*_tet_* promoter (Fig. 2). pKTNTET additionally carries the genes *tetR* encoding the *tet* repressor and *bla* for ampicillin resistance. For convenient subsequent MtCM overproduction, pKTNTET is also equipped with the strong P_T7_ promoter (and an efficient ribosome binding site) more proximal to the MtCM gene, in tandem to P*_tet_* (31). P_T7_-directed gene overexpression for biochemical studies just required retransformation of candidate plasmids into strain KA12 containing plasmid pT7POLTS, which carries the gene for the P_T7_-specific T7 RNA polymerase (31).

**Table 1:** **Comparison of the catalytic parameters of His-tagged and untagged evolved MtCM variants**

Cycle	Protein	Mutations*^a^*	*k*_cat_*^b^* (s^−1^)	*K_m_^b^* (μm)	*k*_cat_/*K_m_^c^* ×10^4^ (m^−1^ s^−1^)	*T*_m_*^e^* (°C)
	MtCM*^f^*		2.0 ± 0.1	1140 ± 90	0.175 ± 0.009	74 ± 0
	MtCM*^f^* + MtDS*^g^*		8.1 ± 1.9	34 ± 3	24 ± 6	-
0	H_6_-MtCM*^g^*	-	nd	>1700	0.094 ± 0.006*^d^*	75 ± 0
I	3p3*^g^*	PD	12 ± 0	570 ± 17	2.1 ± 0.1	81 ± 1
II	3p5*^g^*	PD/PDAM	31 ± 2	380 ± 20	8.3 ± 0.8	82 ± 0
III	re4.7s11*^g^* (= s11)	PD/PDAM, V62I, D72V	23 ± 3	72 ± 7	33 ± 5	>88
IV	s10es4.15*^g^* (= s4.15)	PD/PDAM, V62I, D72V, V11L, D15V, K40Q	14 ± 2	31 ± 7	45 ± 11	82 ± 1
	N-re4.7s11 (= N-s11)	PD/PDAM, V62I, D72V	28 ± 2	62 ± 17	45 ± 13	88 ± 1
	N-s10es4.15 (= N-s4.15)	PD/PDAM, V62I, D72V, V11L, D15V, K40Q	20 ± 4	43 ± 7	47 ± 11	83 ± 1

*^a^* PD/PDAM stands for T52P, V55D, R87P, L88D, G89A, and H90M replacements.

*^b^* CM activity was determined in 50 mm potassium phosphate buffer, pH 7.5, containing 0.1 mg/ml BSA. The disappearance of chorismate was monitored at 310 nm (30 °C). Kinetic parameters were derived by fitting specific initial velocities to the Michaelis-Menten equation (raw data for representative examples are shown in Fig. S6). *k*_cat_ is calculated per active site. Substrate concentration was varied between 20 and 2000 µm; *nd*, not determined because substrate saturation could not be achieved due to high chorismate absorption above 2 mm. Mean and standard deviation (σ_n−1_) were derived from at least two independent biological replicates.

*^c^* Values obtained from error propagation.

*^d^* Value obtained from averaged initial velocities of respective Michaelis-Menten plots.

*^e^* The melting temperature was determined using CD spectroscopy. Raw data and the fitting equation for representative MtCM variants are shown in Fig. S7.

*^f^* Data from the literature (11).

*^g^* Enzyme variants containing an N-terminal His_6_-tag.

### Cassette mutagenesis of MtCM regions that interact with MtDS (cycles I and II)

**Figure 4.**
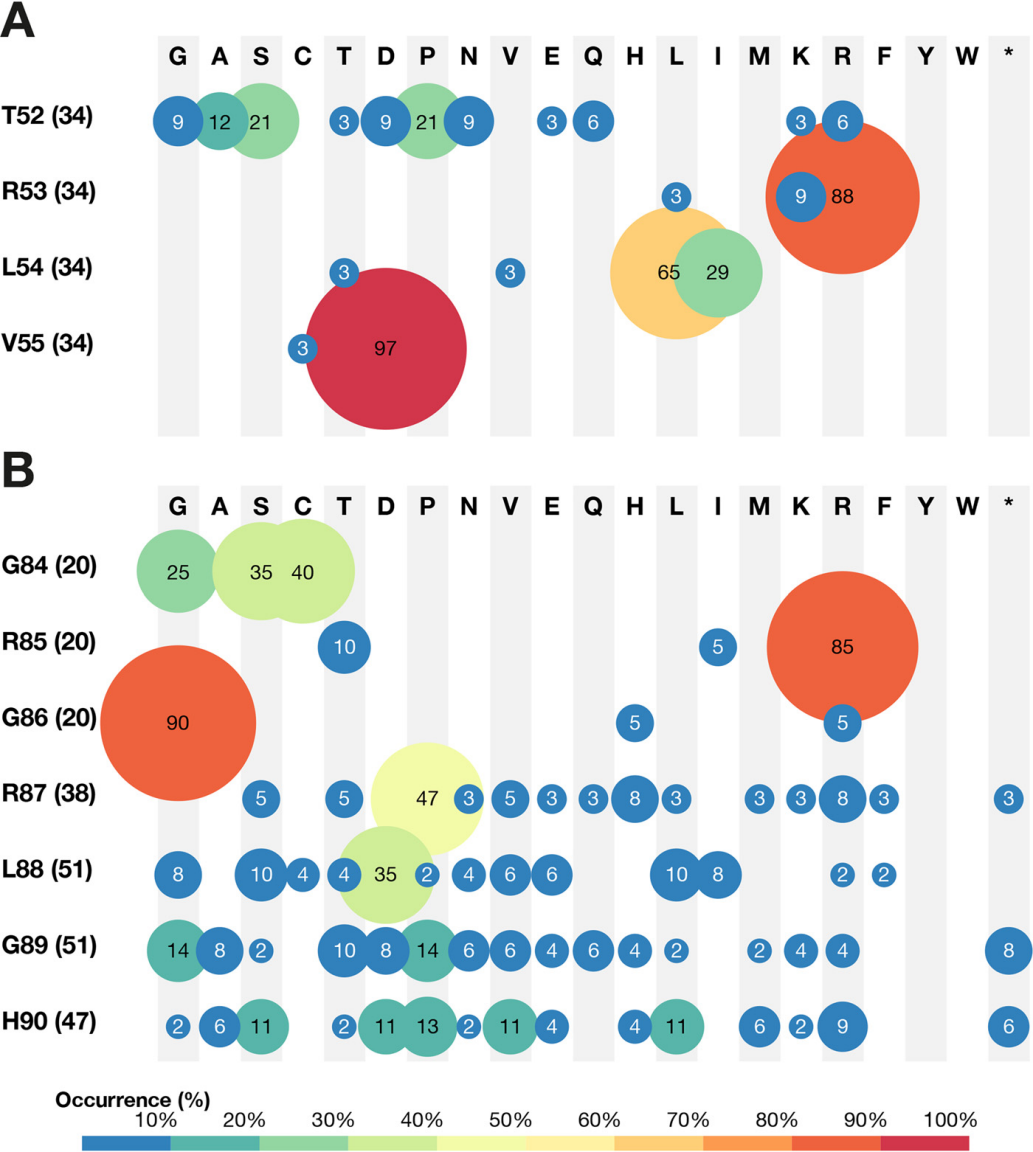
**Selected MtCM sequences from evolutionary cycles I and II.** *A*, results from cycle I of directed evolution. Shown is the amino acid distribution at the randomized positions as derived from sequencing of 34 TRLV library members growing on selective M9c plates. *B*, cycle II sequencing data. Included are 52 variants selected on M9c+pFPhe from libraries CT7, CT-LGH, and CT-RLGH. Circle size and color correlate with the frequency of individual encoded residues with the *color code* shown below the *panels*. Amino acids are listed with their one-letter abbreviation. The percentage of a particular residue at each randomized position is given within the *circle*, and the absolute number of codons considered in the compilations is indicated in *parentheses* next to the WT residue listed on the *left* for each sampled position.

During a first evolutionary cycle (cycle I), the flexible loop between helices H1 and H2 of H_6_-MtCM was targeted by cassette mutagenesis (Fig. 3). This loop, encompassing residues 50 to 55, underwent significant structural changes upon interaction with MtDS resulting in MtCM activation (11) (Fig. 1*C*). Residues Thr^52^, Arg^53^, Leu^54^, and Val^55^ from the H1-H2 loop were randomized via NNK (N = A/C/G/T, K = G/T) codons, and the resulting gene library was transformed into KA12/pKIMP-UAUC to select for active catalysts (Fig. 2). Sequence analysis of the mutant H_6_-MtCM genes from 34 transformants growing after 4 days at 30 °C on M9c minimal medium revealed a remarkable pattern. With the exception of two clones showing Cys, Val^55^ was replaced by an aspartate residue in all selected MtCM variants (Fig. 4*A* and Fig. S1). Positions 53 (Arg) and 54 (Leu) of MtCM were somewhat more variable and tolerated chemically similar replacements. Position 52 (Thr) showed a slight preference for both Pro and Ser instead of the WT Thr in MtCM. Variant PHS08-3p3 (henceforth called 3p3), which combines the two substitutions T52P and V55D, has a 22-fold higher catalytic efficiency (*k*_cat_/*K_m_* = 2.1 × 10^4^ m^−1^ s^−1^) than H_6_-MtCM.

For evolutionary cycle II (Fig. 3), 3p3 was used as a template to randomize C-terminal residues, which are known to be important for the catalytic machinery of MtCM (Gly^84^, Arg^85^, and Gly^86^) or MtDS-mediated activation of MtCM (Arg^87^, Leu^88^, Gly^89^, and His^90^) (11, 31). The three independent libraries PD/LGH, PD/RLGH, and PD/CT7 randomized three, four, and seven positions in the C-terminal region, respectively. Thereby, the codon format NNN allowed for all three translational stop codons to probe for truncated active enzymes (Fig. 3). Indeed, the results suggest that the two to four most C-terminal residues are dispensable in some highly functional variants (Fig. 4*B*). Interestingly, only Cys, Ser, or Gly appear to be allowed at position 84 (Gly in WT MtCM), and the WT residues Arg^85^ and Gly^86^ clearly dominated at the corresponding positions (Fig. 4*B* and Fig. S1).

The best of the characterized catalysts (PHS10-3p5; 3p5) from the PD/RLGH library contained the substitutions R87P, L88D, G89A, and H90M at the C terminus, in addition to the changes selected for in 3p3 (Table S1). Variant 3p5 catalyzed the reaction 4× faster (*k*_cat_/*K_m_* = 8.3 × 10^4^ m^−1^ s^−1^) than 3p3 (Table 1).

### Evolution of inter-subunit destabilized MtCM variants (cycle III)

Despite an already impressive 88-fold increase in catalytic efficiency, variant 3p5 was still 3× less active than MtDS-activated WT MtCM. However, along with the improving CM activity, the suitable dynamic range for selection using the KA12/pKIMP-UAUC–based system was exhausted. Specifically, the tight repression of *aroQ_δ_* gene expression in the absence of the P*_tet_*-inducer tetracycline and an enhanced demand for CM activity on plates lacking both Phe and Tyr, but containing high concentrations of the toxic phenylalanine analog dl-*para*-fluoro-phenylalanine (pFPhe), which efficiently competes with intracellularly produced Phe, no longer showed a growth deficiency (for more details, see Fig. 2 and “Experimental procedures”).

To explore further whether there is an intrinsic limitation to the catalytic machinery of MtCM, the selection stringency first needed to be increased. As a fully complementary approach to manipulating gene expression and lowering protein production, we sought to introduce targeted lesions into the MtCM scaffold to impair the efficiency of variant 3p5 to a level again low enough for effective selection. The application of an analogous perturbation strategy in previous studies had led to catalytically more active and more stable proteins once the lesions were removed (32–35).

For cycle III of the evolutionary experiments, we implemented an MtCM inter-subunit destabilization strategy by mutagenizing Leu^24^ and Leu^31^, the side chains of which point toward the interface formed by the H1 helices of the two subunits (Fig. 3). Replacement of these residues was expected to destabilize the dimer and thus lower the catalytic efficiency of 3p5 as the active site lies at the dimer interface (11). To achieve this, three gene libraries were constructed to fully randomize either Leu^24^ or Leu^31^ or both residues simultaneously via NNK codons. For a fourth library, positions Leu^24^ and Leu^31^ were mutated via the RNK (R = A/G, N = A/C/G/T, K = G/T) codon to exclude stops and at the same time enrich the library for charged residues, because buried charges are expected to effectively destabilize the MtCM structure.

The libraries were individually transformed into KA12/pKIMP-UAUC and grown on weakly selective agar plates (M9c+Phe+Tet^500^; Fig. 2*B*). This allowed the identification of impaired but still active variants that were subsequently verified not to complement the CM defect on higher-stringency agar plates such as M9c+Tet^80^ (Fig. 2*B*). DNA sequence analysis of such weak complementors showed that the buried residues Leu^24^ and Leu^31^ in 3p5 had been replaced by charged (Glu, Arg), polar (Thr, Ser, Gln), bulky (Trp), or small (Gly) amino acids. Kinetic and thermal denaturation assays of several of these 3p5-derived variants revealed that weaker *in vivo* activity correlated with a loss of thermal stability and catalytic efficiency (*k*_cat_/*K_m_*). For instance, a variant of 3p5 with L24R and L31R replacements exhibited a 48 °C lower melting temperature (*T*_m_) and a 40-fold loss in catalytic efficiency. With L24G and L31R substitutions, the *k*_cat_/*K_m_* was even reduced by 170-fold (Table S2), validating our perturbation strategy.

**Table 2:** **Data collection and refinement statistics for the evolved variant N-s4.15**

**Data collection and processing**	
ESRF beamline	BM14
Wavelength (Å)	0.9538
Space group	*P*6_4_
Unit cell parameters	
*a*, *b*, *c* (Å)	54.6, 54.6, 63.2
Resolution (Å)	37.9–1.5 (1.51–1.49)
*CC*_1/2_ (%)	99.9 (33.2)
*I/*σ (I)	15.0 (0.3)
Completeness (%)	95.4 (82.9)
Multiplicity	6.1 (1.9)
No. of unique reflections	16,797 (2358)
**Refinement**	
*R*_work_*/R*_free_ (%)*^a^*	19.0/21.9
**No. of atoms**	
Protein	635
Water	71
***B*-factors (Å^2^)**	
Protein	48.7
Water	60.5
**r.m.s.d. from ideal geometry**	
Bond lengths (Å)	0.020
Bond angles (°)	1.89
**Ramachandran (%)**	
Favored	100
Allowed	0
Disallowed	0
**PDB ID**	5MPV

Values in *parentheses* refer to the highest-resolution shell.

*^a^* $R=\;\sum \left|\left|F_{o}\right|-\left|F_{c}\right|\right|/\sum \left|F_{o}\right|$ where *F*_o_ and *F*_c_ are the observed and calculated structure factors, respectively. *R*_free_ is *R* calculated for 5% randomly selected reflections, which were omitted from the refinement.

Several catalytically impaired inter-subunit variants were chosen as independent parents for cycle III of directed evolution to offer a range of starting points for further improvement (Fig. 3 and Table S2). In addition to the 3p5 mutations, the templates individually contained the destabilizing substitutions L24E, L24G/L31R, L24R/L31R, L24Q/L31G, L24W/L31T, or L24T/L31S (Fig. 3). Error-prone PCR (epPCR) (36) and DNA shuffling (37) were employed to randomize the corresponding genes. The latter technique also offered the possibility to mix in genes carrying the previously identified beneficial mutations encoding G43V, V62I, R82Q, and D75Y (38), in addition to T52P, V55D, R87P, L88D, G89A, and H90M from 3p5. Both mutagenesis strategies targeted the gene region downstream of codon positions 24 and 31 to exclude reversion of the destabilizing mutations back to the WT residues.

From each of the 12 resulting libraries, several clones growing under stringent conditions were sequenced, revealing that the segment between Glu^37^ and Ala^45^ was most heavily mutagenized (Fig. S2). This stretch of residues is directly adjacent to the catalytic residue Arg^46^. Importantly, the replacements T52P and V55D introduced during the first round of directed evolution were retained, whereas several C-terminal substitutions selected during cycle II were again altered in individual selected variants. Kinetic analysis of 32 further-evolved destabilized enzymes showed mostly increased or similar catalytic efficiencies *k*_cat_/*K_m_* (Table S3) compared with their direct precursors. Their *k*_cat_/*K_m_* values were further augmented when the destabilizing mutations at positions 24 and 31 were reverted to the corresponding WT Leu residues (Table S4). The most active variant re4.7s11 (abbreviated as s11), having V62I and D72V replacements additional to those in 3p5, exhibited a 4-fold improved *k*_cat_/*K_m_* of 3.3 × 10^5^ m^−1^ s^−1^. Even though this value already surpassed the catalytic activity measured for MtDS-activated MtCM, variant s11 was chosen as the parent for another round of directed evolution.

### Evolution of terminally truncated MtCM variants (cycle IV)

Also for cycle IV, we invoked a perturbation-compensation strategy (Fig. 3). This time, a terminal truncation approach was applied to destabilize s11, the top variant from cycle III, as exemplified by Hecky *et al.* (33, 35, 39) for evolving thermostable β-lactamases through rounds of truncation, optimization, and re-elongation. The removal of terminal residues often causes severe folding and structural perturbations and may even turn a protein into a molten globule (40). We hypothesized that MtCM might be sensitive to C-terminal truncation because these residues play a crucial role in MtDS-based activation (11, 31), and C-terminal substitutions clearly improved the activity of the 3p3 variant (see cycle II above and Table S1). In addition, we truncated the N terminus based on the observation that exchanging the first four N-terminal residues with the His-tag reduced the catalytic efficiency of WT MtCM nearly by half (Table 1). Because variant s11 was already highly active, we anticipated that simultaneous truncations at both the N and C termini would be required to reach a sufficiently low activity level again amenable for effective *in vivo* selection. Our terminal truncation approach allowed exploration of the MtCM sequence space located N-terminal from positions 24 and 31, which was spared in the previous evolutionary cycle.

A summary of the truncation-destabilization strategies applied to variant s11 in cycle IV is depicted in Fig. 3. The strategies encompassed (i) incremental two-residue deletions at the N terminus up to Arg^18^, (ii) successive truncations at the C terminus until Arg^85^, and (iii) randomization of residue Arg^85^. For the LdNdC library, options (i) and (ii) were combined. For library LdNR85X, N-terminal deletions according to strategy (i) were combined with option (iii) in a five-residue C-terminally truncated version of s11. As for the inter-subunit destabilized variants in cycle III, we looked for weakly active truncated clones unable to grow at high stringencies. From library LdNdC, only variant dNdCs1, which had the maximum allowed deletion of 10 N-terminal and five C-terminal residues, fulfilled these criteria. In contrast, library LdNR85X yielded several compromised variants due to mutagenesis of Arg^85^. Characterization of truncated s11-derived enzymes showed that they lost at least two orders of magnitude in *k*_cat_/*K_m_* (Table S5). Moreover, proteins with deletions in the N-terminal region were less stable, as reflected by a decrease in *T*_m_ (*e.g.* for variant dNdCs1 by at least 14 °C).

The fourth and most stringent cycle of directed evolution built on the severely impaired truncated MtCM variants dNdCs1, dNdCs4, and dNdCR85Xs10 (Table S5). Their genes were mutagenized by epPCR (for dNdCs4 also by DNA shuffling in combination with a RepA protein degradation tag; details under “Experimental procedures”), and the corresponding three random libraries were subjected to selection on highest-stringency plates (Fig. 2*B*). Sequencing of winner clones selected from the three ultimate libraries revealed that the stretch between Ala^36^ and Ala^45^ acquired particularly many new mutations (Fig. S3). This was already observed for variants evolved during cycle III (Fig. S2), suggesting that these replacements were generally beneficial regardless of the destabilization mode.

We assessed the *in vitro* activities of variants emerging from the final cycle IV libraries both before (Table S6) and after the deleted termini were added back (Table S7). About half of the truncated variants selected *in vivo* showed a 2–18-fold higher *k*_cat_/*K_m_ in vitro* when compared with their truncated parents (Table S6). Moreover, after re-elongation of the termini, the variants selected from the gene libraries had typically gained two to three orders of magnitude in *k*_cat_/*K_m_* (Table S7). However, compared with the winner of cycle III (s11), the improvement is modest at best. The top variant s10es4.15 (subsequently referred to as s4.15) contained mutations V11L, D15V, and L40Q and had a *k*_cat_/*K_m_* (4.5 × 10^5^ m^−1^ s^−1^, Table 1), which is only 1.4-fold higher than that of s11.

### Biochemical characterization of key evolutionary intermediates

The biochemical data for the best variants from each evolutionary cycle revealed that the evolved enzymes possessed not only improved catalytic efficiencies *k*_cat_/*K_m_* but also higher thermal stabilities relative to the starting WT (H_6_-MtCM; Table 1). The largest catalytic improvement (22-fold) was obtained for variant 3p3 after the first evolutionary cycle as a result of substitutions T52P and V55D in the H1-H2 loop of MtCM. The contribution of the individual replacements was assessed after subcloning, revealing that T52P alone ameliorated *k*_cat_/*K_m_* by 6-fold whereas the V55D substitution on its own yielded a 12-fold enhancement (Table S1). Cycle II yielded variant 3p5 with the substitutions R87P, L88D, G89A, and H90M at the C terminus. The improvement of *k*_cat_/*K_m_* (4-fold relative to 3p3) was achieved by both a reduction in *K_m_* and an increase in *k*_cat_. Introduction of mutations V62I and D72V into variant s11 during cycle III improved substrate binding by reducing *K_m_* 5.3-fold, elevating *k*_cat_/*K_m_* by another factor of 4. The fourth and last cycle of evolution resulted only in minor gains. The overall activity of the best variant s4.15 was slightly improved, mainly by lowering *K_m_* by 2.3-fold, now matching the *K_m_* of MtDS-activated WT MtCM. The *k*_cat_ and *K_m_* tradeoff exhibited by this variant suggests that the additional mutations V11L, D15V, and K40Q helped to stabilize the enzyme-substrate complex rather than the transition state of the CM reaction.

When equipping WT MtCM and the evolutionary intermediates with a His-tag to facilitate quick access to the proteins, we replaced the four native N-terminal residues (MNLE) of MtCM (Fig. 1*E*) by the sequence MHHHHHHSSG, thereby reducing the catalytic efficiency of the native protein to half (Table 1). Thus, to probe the benefit of the evolutionarily acquired mutations in the native format, the original N terminus was restored for the best variants. As shown in Table 1, the corresponding construct N-s4.15 retained the high catalytic efficiency of s4.15, and the activity of the non-His-tagged variant N-s11 was augmented to the level of N-s4.15. The untagged versions still displayed a high thermal stability (Table 1).

We also investigated whether MtDS was still able to activate the CM activity of the evolved variants N-s11 and N-s4.15 as it does for WT MtCM. The kinetic data demonstrated that the activation potential was completely lost in the new variants (Table S8). This is not unexpected because they already had very high CM activity and because the H1-H2 loop and the C terminus, known to interact with MtDS (11, 31), were heavily mutated. In fact, activation by MtDS was found to be impaired by 30-fold in previously reported MtCM variants lacking the two C-terminal residues (31).

### Impact of activity-boosting substitutions on the overall MtCM fold

**Figure 5.**
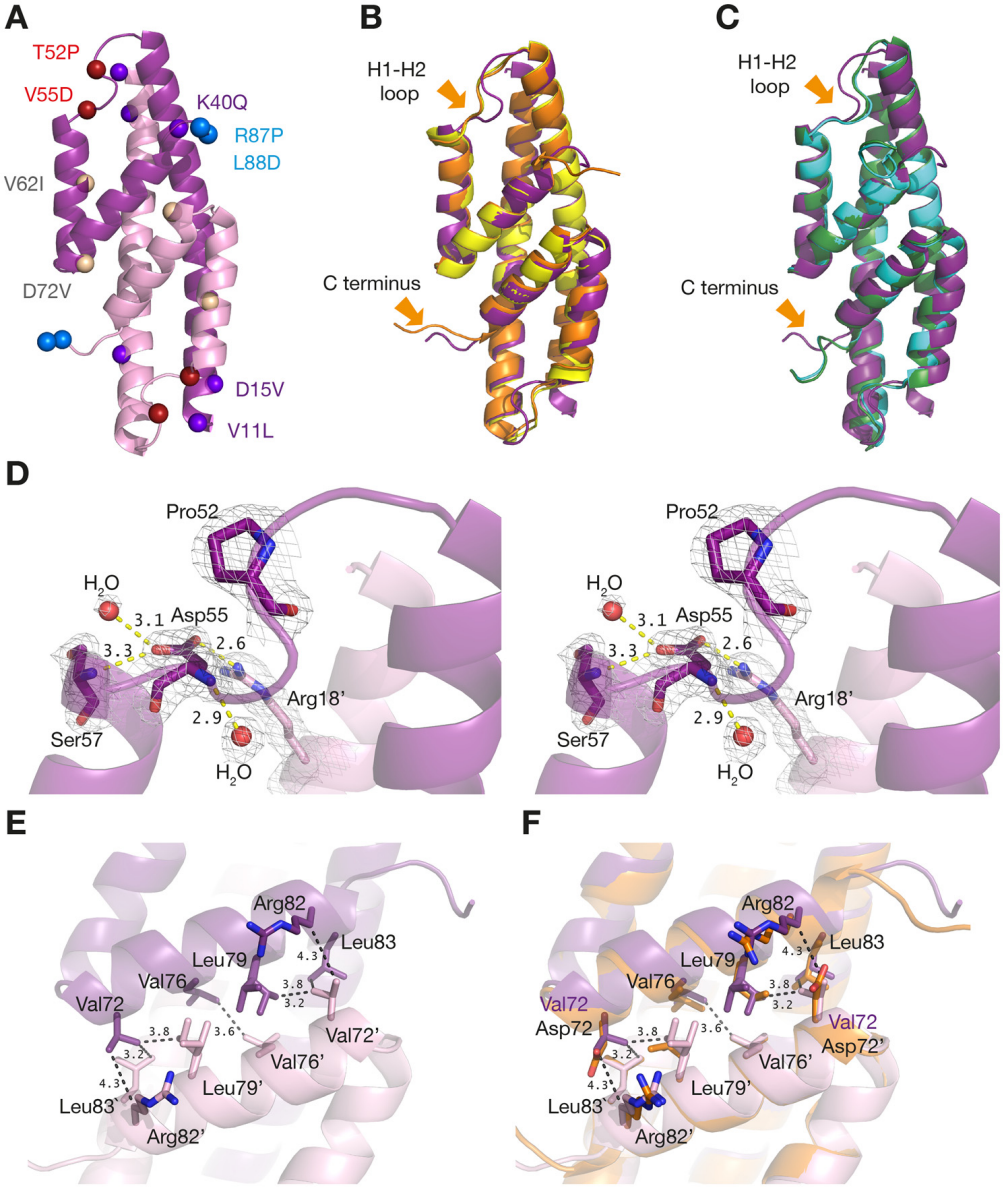
**Structure of top-evolved variant N-s4.15 and comparison with native MtCM.** *A*, overall structure of N-s4.15. The two subunits are colored in different *shades of purple*. *Colored spheres* locate the mutations identified during the course of directed evolution. *B*, superimposition of N-s4.15 (*purple*) with malate-bound (*orange*; PDB ID: 2VKL) and apo (*yellow*; PDB ID: 2QBV) MtCM WT structures. *C*, superimposition of N-s4.15 (*purple*) with MtDS-bound activated MtCM with (*green*; PDB ID: 2W1A) or without (*cyan*; PDB ID: 2W19) TSA (ligand not shown). The largest structural differences are pointed out with *arrows*. Note that there are crystal contacts in both of these regions (see Fig. S5). *D*, close-up stereo view of the H1-H2 loop of N-s4.15. The electron density is contoured at 1σ. Pro^52^ induces a kink in the H1-H2 loop and Asp^55^ N-caps helix H2. Polar interactions of Asp^55^ (distance in Å) are indicated with *yellow dashed lines*. *E*, position of Val^72^ at the N-s4.15 dimer interface. Prominent van der Waals interactions are indicated by *black dashed lines*, and relevant residues from the other protomer are denoted by a prime (′). *F*, N-s4.15 dimer interface as in *E* superimposed with malate-bound (unactivated) MtCM (*orange*, with the WT Asp^72^; PDB ID: 2VKL). The structure of N-s4.15 is represented by PDB ID: 5MPV (this work) in all panels.

To elucidate the structural basis for the boost in catalytic activity, we solved the crystal structure of the top variant N-s4.15 to 1.5 Å resolution (Table 2). The protein adopts the typical homodimeric six-helical fold of AroQ_δ_ enzymes (Fig. 5*A*) and aligns well overall with the two MtCM WT structures deposited in the PDB (PDB ID: 2QBV (41) and PDB ID: 2VKL (11); Fig. 5*B*).

When WT MtCM is activated through complex formation with MtDS, it undergoes several pronounced conformational changes, particularly at the H1-H2 loop and the C terminus (Fig. 1*C*). As a result, several active site residues are brought into catalytically competent conformations, yielding a 100-fold activity increase (11). The crystal structure of the most highly evolved variant N-s4.15 allows for a direct comparison with the conformation of WT MtCM in the MtDS-activated complex (Fig. 5*C*), enabling further insights into the activation mechanism.

Two substitutions that strongly enhanced the autonomous catalytic efficiency of MtCM are T52P and V55D in the H1-H2 loop, which are the sole substitutions in the intermediate variant 3p3. Interestingly, a proline and an aspartate residue are also present in structurally equivalent positions in the naturally efficient enzymes EcCM and *MtCM (Fig. 1*E*). In N-s4.15, this loop adopts a similar conformation as in EcCM, *MtCM, and MtDS-complexed MtCM that is distinct from the loop conformation in free MtCM (Fig. 5, *B* and *C* and Fig. S4). The activated loop is highly kinked, either because of Pro^52^ in the evolved variant (Fig. 5*D*) or induced by binding to MtDS (11) (Fig. 1*C*). A serine residue can induce a similar kink when its side chain bends back to interact with the main chain nitrogen, possibly explaining why serine also featured prominently at position 52 after evolutionary cycle I (Fig. 4*A*). Asp^55^ ensures the return to the helical conformation at the C-terminal side of the H1-H2 loop by N-capping helix H2 (Fig. 5*D*). This residue further engages in a network of polar interactions with the backbone of Ser^57^ and the guanidinium group of Arg^18^, a catalytic residue provided by the other MtCM protomer.

In the native system, docking of the C terminus of MtCM onto MtDS determines the conformation of the loop connecting helices H1 and H2 (11). Interaction of the C-terminal carboxylate with Arg^53^ and concomitant C-terminal reorientation exposes Leu^88^ to the H1-H2 loop, where it interacts with Leu^54^, causing a register shift in residues 54 and 55 close to the active site (11). In many evolved MtCM variants, including 3p5 and s4.15, Leu^88^ is replaced with Asp^88^, which, because of its negative charge, cannot engage in equivalent interactions. Nevertheless, residues 54 and 55 adopt similar conformations in the two activated MtCM structures, but for different reasons. Rather than relying on C-terminal contacts, the catalytically optimal conformation of the H1-H2 loop is enabled by the T52P and V55D substitutions in the highly evolved variants.

The catalytic efficiency of MtCM was further enhanced by replacements at the C terminus (RLGH to PDAM), as exemplified in variant 3p5, which had evolved in cycle II. Also here, a proline residue (at position 87) featured prominently among the selected variants (Fig. 4*B*). The crystal structure of N-s4.15 shows that Pro^87^ introduced another kink, resulting in reorientation of the C terminus (Fig. S5). This rearrangement is reminiscent of the conformational change induced upon complex formation with MtDS, where the C terminus of MtCM docks into a hydrophobic groove of its binding partner, boosting CM activity by 100-fold (11). However, in the variant N-s4.15, the C-terminal residues are less well-ordered compared with MtDS-activated WT MtCM (average *B*-factors of Pro^87^ and Asp^88^ are >100 Å^2^), and beyond residue 88, hardly any electron density is visible, suggesting that the C terminus is flexible in solution. In N-s4.15, Pro^87^ and Asp^88^ adopt conformations that are significantly different from both free and MtDS-bound MtCM structures (Fig. 5, *B* and *C* and Fig. S5). The differences in the C-terminal region between uncomplexed WT enzyme and N-s4.15 (Fig. 5*B*) are in part due to the kink caused by Pro^87^ but also reflect distinct crystal contacts imposed by the different space groups (*P*4_3_2_1_2 and *P*6_4_, respectively; Fig. S5).

Final tweaks in catalytic optimization were achieved by implementing perturbation-compensation strategies (Fig. 3). Inter-subunit destabilization and subsequent directed evolution (cycle III) introduced substitutions V62I and D72V into variant 3p5. Val^72^ is located at the dimer interface and tightly packed into a hydrophobic pocket provided by Leu^79^, Leu^83^, and the hydrophobic part of the Arg^82^ side chain from the other protomer (Fig. 5*E*). The increase in melting temperature caused by replacing Asp^72^ with the uncharged valine (Fig. 5*F*) in s11 (Table 1), along with isoleucine instead of Val^62^ (see below), can probably be explained by a more compact packing. The V11L, D15V, and K40Q replacements, which were acquired during the ultimate evolutionary cycle, jointly increased the overall activity slightly, predominantly by lowering *K_m_* (Table 1). Examination of the unliganded N-s4.15 structure suggests that the effect of these changes on substrate binding is indirect despite their proximity to the active site (Fig. 5*A*).

### Active site of top-evolved variant N-s4.15

**Figure 6.**
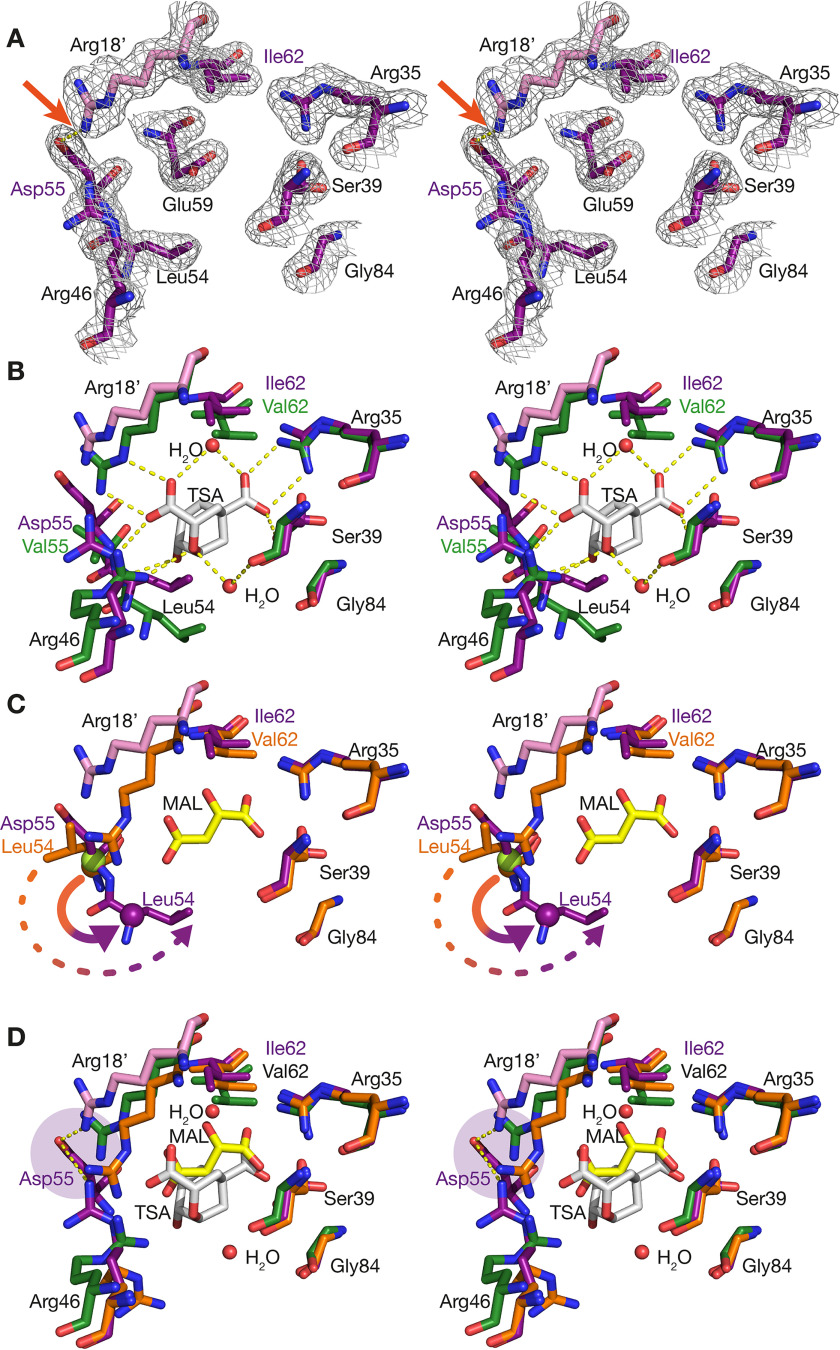
**Stereo images of the active site of MtCM.** The structure of the top-evolved variant N-s4.15 (PDB ID: 5MPV, this work) is shown in *purple/pink*. Some residues (*e.g.* Glu^59^) were omitted for clarity in individual panels. *A*, N-s4.15 active site with corresponding 2*mF*_o_-*DF*_c_ electron density (*gray mesh*), contoured at 1σ. The hydrogen bond between residues Asp^55^ and Arg^18^ (prime) of the other subunit is highlighted by an *arrow*. *B*, superimposition of N-s4.15 and MtDS-activated WT MtCM (*green*; PDB ID: 2W1A) in complex with TSA (*white sticks*), including relevant water molecules and H-bonds. *C*, superimposition of N-s4.15 and WT MtCM (*orange*; PDB ID: 2VKL) bound to malate (*yellow*). A register shift in the corresponding H1-H2 loops is apparent from the identical location of the C_α_ atoms of Leu^54^ of WT MtCM (*orange sphere*) and Asp^55^ of N-s4.15 (*green sphere on top*). It is also visualized by the relocation of the *orange Leu^54^ C_α_ sphere* to the corresponding *purple C_α_* in N-s4.15 (*solid arrow*) with a concomitant shift of the Leu^54^ side chain by ∼8 Å (tip to tip; *dashed arrow*). *D*, superimposition of N-s4.15 with TSA-bound MtDS-activated (*green*; PDB ID: 2W1A) and malate-bound WT MtCM (*orange*; PDB ID: 2VKL) with a focus on the position of the guanidinium group of the catalytic residue Arg^46^. For the evolved variant N-s4.15, relevant H-bonds with Asp^55^ are shown (*purple disk*).

The conformational changes induced by substitutions T52P and V55D directly affect the active site, which for N-s4.15 (Fig. 6*A*) is very similar to the conformation of WT MtCM when in complex with MtDS (Fig. 6*B*). Of particular importance for the increased catalytic competence of the enzyme is the induced register shift of MtCM residues 54 and 55 in the H1-H2 loop (Fig. 6*C*). In its activated conformation, residue 55 forms a hydrogen bond from its backbone NH to the hydroxyl group of the ligand (Fig. 1*D*). This interaction requires the precise alignment of the backbone amide with the active site, irrespective of the identity of residue 55 (Val or Asp). Interestingly, the Asp^55^ side chain of N-s4.15 engages in a hydrogen bond with Arg^18^ from the other protomer across the active site (Fig. 5*D* and Fig. 6*A*), a catalytic residue that aligns well with the corresponding active conformation in the MtDS complex (Fig. 6*B*). Together with the observation that all V55D variants possess a lowered *K_m_* (Table 1), the structural comparison suggests that positioning of Arg^18^ of the other protomer by Asp^55^ may favor substrate binding.

The most important catalytic residue, Arg^46^, must interact directly with the ether oxygen of TSA (Fig. 1*D*) to stabilize the developing negative charge in the transition state of the CM reaction (11, 17). In native uncomplexed MtCM, the Arg^46^ side chain points away from the active site (Fig. 6*D*). It is restrained in this catalytically unproductive conformation by interaction (through Nη_1_) with the backbone oxygen of Gly^51^ (Fig. S4), a residue close to the register shift described above. Whereas the guanidinium group of Arg^46^ in the evolved N-s4.15 is still not in an ideal position but is pulled toward the newly introduced acidic residue Asp^55^ (Fig. 6*D*), it is more similar to that of MtDS-activated MtCM compared with native uncomplexed MtCM (Fig. S4). It is conceivable that productive ligand binding to the active site will attract Arg^46^ to the negatively charged substrate, resulting in a catalytically competent geometry.

The V62I substitution (together with D72V, Fig. 5) resulted in a 5-fold improved *K_m_* of the evolutionary intermediate s11 (Table 1). Ile^62^ assumes a conformation that is essentially identical to Val^62^ in the WT (Fig. 6*C*), but the additional methyl group of Ile^62^ may enable tighter packing with the ligand. Indeed, this extension reaches into the space occupied by a methyl group of Val^62^ after its backbone C_α_ is repositioned as a consequence of MtCM activation by MtDS (Fig. 6*D*).

Of the remaining active site residues (Fig. 1*D*), Arg^35^, Ser^39^, and Arg^58^ superimpose quite well with their counterparts in the active MtCM-MtDS complex (PDB ID: 2W1A (11); Fig. 6, *B* and *C*; Arg^58^ not shown), whereas Glu^59^ in N-s4.15 adopts a conformation that would sterically clash with a ligand in the active site (Fig. 6, *A* and *B*). The position of its side chain seems to help balance the many positive charges in the apo enzyme (Fig. 6*A*). Glu^59^ could, however, easily reorient upon ligand binding to adopt a catalytically favorable conformation. In the active MtCM-MtDS complex (PDB ID: 2W1A) (11), Glu^59^ engages in a hydrogen bond with the hydroxyl group of TSA (Fig. 1*D*). It is fixed in this position by a bidentate salt bridge with Arg^85^, which is appropriately reoriented through the interaction of C-terminal MtCM residues with MtDS (11).

Overall, most active site residues of N-s4.15 adopt conformations resembling the ones observed in the MtDS-activated WT MtCM. They are often preorganized to assume catalytically competent positions for ligand binding and catalysis of the CM reaction in the absence of MtDS, exhibiting similarities with other naturally efficient CMs, such as EcCM and *MtCM.

## Discussion

In this work, we subjected the mediocre MtCM to four cycles of directed evolution to explore the catalytic potential of this natural enzyme (Fig. 3). We have applied a powerful CM selection system that couples the enzyme's activity to bacterial viability under stringent selective conditions to efficiently identify mutations that improve the catalytic prowess of MtCM. However, the limitations of the original selection approach were already reached after cycle II, when the target enzyme had gained enough proficiency to fully complement the metabolic defect of the selection strain. To increase the selection stringency for the following rounds, we successfully implemented structural perturbation-compensation strategies. These consisted of temporarily crippling the catalytic activity of the evolving MtCM variants followed by augmenting it again through the introduction of new and different mutations. Removal of the deliberately installed lesions in the further-evolved (destabilized) variants resulted in most cases in better catalysts than the starting points.

In the course of the directed evolution experiments we identified MtCM variant s4.15, which is, with a *k*_cat_/*K_m_* of 4.7 × 10^5^ m^−1^ s^−1^, twice as efficient as the MtDS-activated WT enzyme. The crystal structure of N-s4.15 implies that its improved activity is due to a combination of pre-positioning active site residues for efficient substrate and transition state binding, tighter packing of the active site, and an overall stabilization of the fold, as reflected by the increase in the enzyme's melting temperature.

Of all the beneficial substitutions, the introduction of Pro^52^ and Asp^55^ had the biggest effect by reshaping the catalytically important H1-H2 loop. The induced register shift of residues 54 and 55 places the backbone of residue 55 in an optimal position to interact with the ligand's hydroxyl group, whereas the Asp^55^ side chain may pre-align Arg^18^ of the other protomer for better substrate binding. Interestingly, the change from ^52^TRLV^55^ to ^52^PRLD^55^ resembles the corresponding sequences in the AroQ_α_ subclass EcCM (^45^PVRD^48^), the AroQ_γ_ subclass *MtCM (^66^PIED^69^), and the AroQ_β_ subclass ScCM, which has Pro^174^ and Asn^194^ at the homologous positions (Fig. 1*E*). The latter three CMs achieve high catalytic efficiencies without DS interactions, suggesting that having Pro^52^ and Asp^55^ in the H1-H2 loop is an important feature of an autonomously proficient catalytic machinery. In contrast, the fact that native MtCM and many other δ-subclass CMs (11, 31) use Thr^52^ can be rationalized by structural arguments. Threonine (like the frequently selected serine; Fig. 4*A*) can adopt a similar conformation as proline by H-bonding to the main chain nitrogen. In contrast to proline, this conformation is, however, not permanently fixed for threonine (or serine), allowing for greater conformational sampling. Such a temporary “kink-potential” is probably crucial for DS-dependent activity switching of AroQ_δ_-subclass CMs (1, 2, 11).

In N-s4.15, the conformation of the H1-H2 loop enforced by Pro^52^ and Asp^55^ might, upon substrate binding, favor appropriate positioning of Arg^46^ for electrostatic catalysis (17) like in MtDS-activated MtCM (11). However, complex formation with MtDS provides this active conformation by a different mechanism than in autonomous N-s4.15. Rather than taking advantage of an ideally prearranged H1-H2 loop, native MtCM involves its C terminus. By hooking onto MtDS, the C terminus exposes Leu^88^, which in turn binds to Leu^54^, inducing a register shift that extends to residue 55, and concomitantly repositions Arg^46^ for catalysis (11).

The diffusion barrier is generally regarded as the only obvious limitation for the *k*_cat_/*K_m_* of “perfect” enzyme catalysis (42). However, whereas some enzyme-catalyzed reactions (43, 44) reach the diffusion-limited apparent second-order rate constant (*k*_cat_/*K_m_*) of 10^8^–10^9^ m^−1^ s^−1^, a global analysis (45) of catalytic parameters revealed that the “average enzyme” only has a *k*_cat_/*K_m_* of ∼10^5^ m^−1^ s^−1^. It is of significant interest to elucidate the factors behind nature's reasons for making seemingly less-than-perfect catalysts: is it the difficulty of the chemistry, or the intrinsically limited potential of proteins to evolve for a given catalytic task, or the lack of sufficient selection pressure?

**Figure 7.**
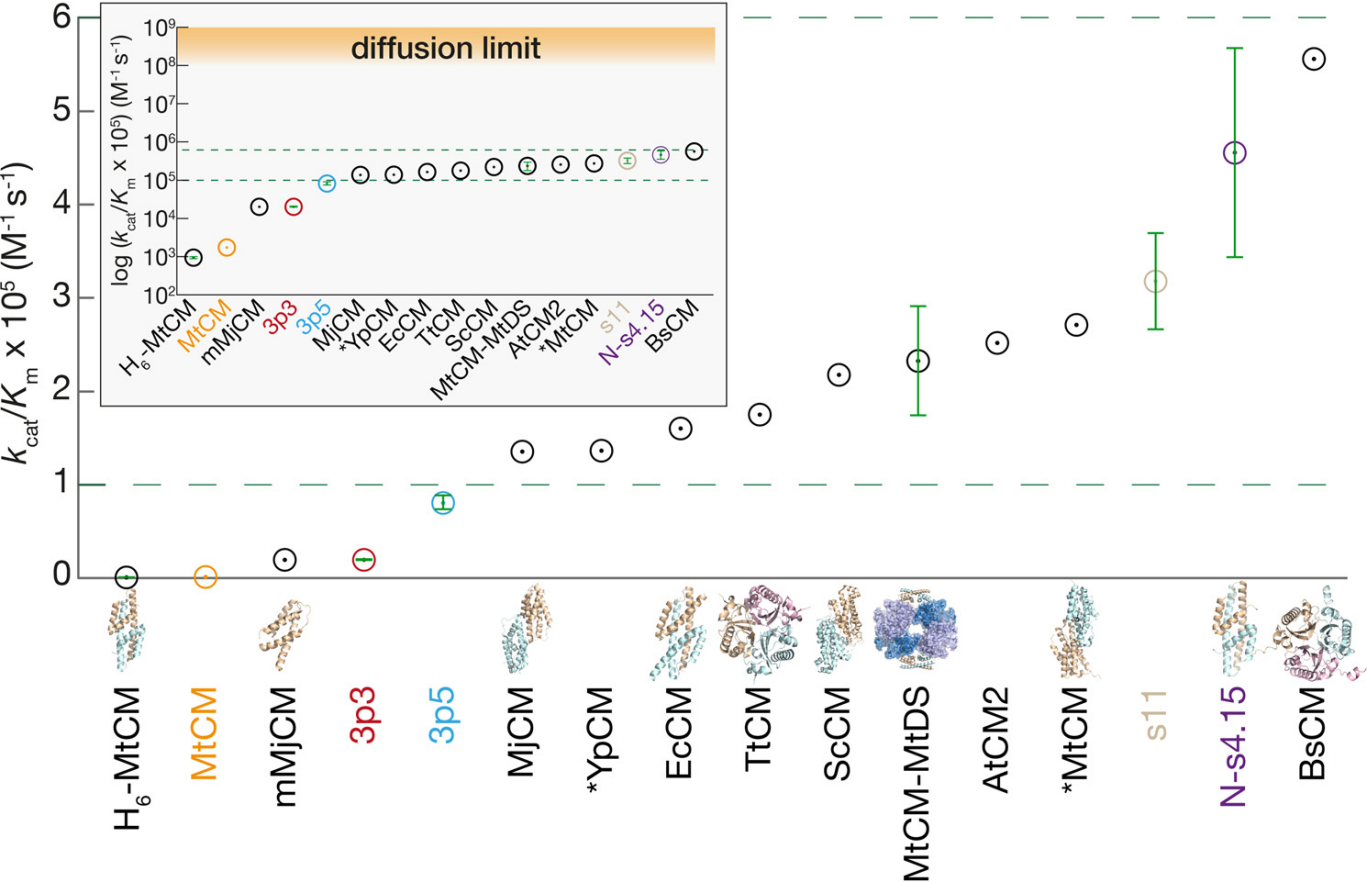
**Catalytic efficiencies of evolved MtCM variants compared with natural CMs.** The range of *k*_cat_/*K_m_* values typically measured for natural CMs is delineated by two *green dashed lines* both in a linear and logarithmic (*inset*) representation. The corresponding kinetic parameters and their references are provided in Table S9. Key variants evolved in this work are color-coded according to the evolutionary scheme of Fig. 3 and provided with experimental *error bars*. Where available, the protein structures are displayed for illustration (not drawn to scale), including MtCM (PDB ID: 2VKL, (11)), mMjCM (an engineered monomeric *Methanocaldococcus jannaschii* CM; PDB ID: 2GTV, (78)), *YpCM (secreted *Yersinia pestis* CM; PDB ID: 2GBB, (41)), EcCM (PDB ID: 1ECM, (13)), TtCM (*Thermus thermophilus* CM; PDB ID: 1UI9), ScCM (PDB ID: 1CSM, (15)), MtCM-MtDS (PDB ID: 2W19, (11)), *MtCM (secreted *M. tuberculosis* CM; PDB ID: 2FP1, (20)), N-s4.15 (PDB ID: 5MPV, this work), and BsCM (*Bacillus subtilis* CM; PDB ID: 1DBF, (79)).

Even though the activity of our top-evolved MtCM variant N-s4.15 is still 2–3 orders of magnitude below the diffusion limit, it has reached essentially the same activity level as the best natural CMs characterized so far (Fig. 7). A similar upper value for *k*_cat_/*K_m_* was recently confirmed for a set of previously unknown CMs that were sampled from 1130 natural AroQ sequences of phylogenetically widely diverse organisms (46). The systematic decrease of improvements per evolutionary round and the inability to go beyond ∼10^6^ m^−1^ s^−1^ despite applying sophisticated evolutionary strategies might indicate that we have approached an intrinsic threshold for the evolution of the enzymatic Claisen rearrangement of chorismate.

This interpretation is supported by a recent site-directed mutagenesis study of MtDS, the partner enzyme of MtCM. Guided by statistical coupling analysis, Parker and co-workers (47) generated an MtDS variant (Y131A) impaired in allosteric regulation by the pathway's end products Phe, Tyr, and Trp. Moreover, this MtDS variant lent an unexpected activity boost to WT MtCM, with a reported catalytic efficiency *k*_cat_/*K_m_* of 7 × 10^5^ m^−1^ s^−1^ for MtCM when in the large heterooctameric complex, matching the level for the evolved stand-alone MtCM variant N-s4.15 (47). The origins of this activity enhancement still need to be elucidated, as MtDS residue 131 is positioned ∼30 Å from the MtCM-MtDS interface. It was speculated that tweaked subunit positioning and conformational changes at the interface and/or an altered dynamic equilibrium might have affected the catalytic parameters (47).

That *k*_cat_/*K_m_* of typical natural CMs hardly reaches 10^6^ m^−1^ s^−1^ prompted several investigations into the nature of the rate-limiting steps. Kinetic isotope effects determined for an AroQ_α_-subclass CM and the structurally unrelated AroH-class BsCM (Fig. 7) showed that the chemistry of the [3,3]-sigmatropic rearrangement of chorismate is largely rate-determining for BsCM (48) but not for the AroQ_α_ enzyme (49). The AroQ_α_ result implies a kinetically significant transition state prior to the chemical step, possibly involving ligand complexation or protein conformational changes. Viscosity-variation experiments established that diffusive processes also partially limit the reaction rate of BsCM (50). The fact that its *k*_cat_/*K_m_* is still far below the diffusion limit suggests that a rare conformation of the flexible chorismate and/or the enzyme is required for a catalytically productive binding event (50, 51).

In addition to the intrinsic physicochemical limitations (45) for the Claisen rearrangement of chorismate, evolutionary scenarios must be considered, too. Global data analysis suggests that evolutionary pressure causes natural enzymes to become more proficient until a balance is reached between an organism's metabolic needs and the cost for producing and improving a catalyst (45). As a result, most enzymes found in nature are mediocre rather than functioning at the maximum catalytic efficiency (52). Moreover, natural CMs have evolved to work optimally under physiological conditions including, for instance, the presence of matching upstream and downstream enzymatic processes or the actual substrate and product concentrations within a cell. Because our selection system depends on prevailing intracellular constraints, we speculate that the top-evolved variants have reached a level of catalytic proficiency that would be difficult to exceed by further *in vivo* evolution.

In fact, the MtCM-derived top-evolved N-s4.15 rivals the highest-ever reported CM efficiencies even with its very simple AroQ_δ_ fold, built from the shortest primary sequences known for CMs. At the same time, the evolution experiment demonstrated that the compromised natural enzyme MtCM already possesses all functional groups required for efficient catalysis despite lacking otherwise absolutely conserved active site residues. The relatively facile evolutionary trajectory to boost activity by 2–3 orders of magnitude implies that AroQ_δ_ subclass CMs on their own evolved to be intentionally poor natural catalysts for enabling inter-enzyme allosteric regulation by switching between mediocre and highly active states (1, 2, 11, 22, 23). We expect that analogous rigorous evolutionary studies of natural enzymes will elucidate capabilities and limitations of numerous biocatalysts and shed light on yet undiscovered allosteric control mechanisms. Furthermore, the ease of finding beneficial mutations gives fundamental insights into the development of drug resistance and the evolution of enzyme function.

## Experimental procedures

### Materials and general procedures

Plasmid DNA purification from *E. coli* cultures was performed using the Genomed Jetquick spin columns (Brunschwig AG, Switzerland), NucleoSpin cups (Macherey-Nagel, Germany), or ZR-Miniprep Classic kit (Zymo Research). DNA from PCR, restriction digestions, and ligations was purified either directly from the reactions (using the DNA Clean and Concentrator kit-5 from Zymo Research, Jetquick Spin columns from Genomed, or NucleoSpin cups from Macherey-Nagel) or after agarose gel electrophoresis in TAE (40 mm Tris base, 20 mm acetic acid, and 1 mm EDTA, pH 8.25) buffer (using the Zymoclean^TM^ Gel DNA Recovery kit from Zymo Research or Jetquick Spin columns from Genomed). DNA concentration was determined spectrophotometrically using NanoDrop (Thermo Fisher Scientific). DNA manipulations were performed using standard procedures (53) or according to manufacturer recommendations. All cloned PCR–amplified fragments were checked for undesired mutations by sequence analysis. Sanger DNA sequencing and oligonucleotide synthesis were generally performed by Microsynth AG (Switzerland). Oligonucleotides were purified by desalting for routine primers and by HPLC for degenerate primers by Microsynth AG. Restriction endonucleases, Phusion DNA-polymerase, and T4 DNA ligase (for 16-h library ligations at 16 °C) were purchased from New England Biolabs. pFPhe was from Bachem Holding AG (Switzerland). Chorismate for enzymatic assays was produced following a published protocol (54). Other chemicals were purchased from Sigma-Aldrich/Fluka.

### Bacterial strains and plasmids

*E. coli* strain KA12 (F^−^, λ^−^, Δ*(srlR-recA)306*::Tn*10* (Tet^R^), Δ(*pheA-tyrA-aroF*), *thi-1*, *endA1*, *hsdR17* (r_K-_, m_K+_), Δ(*argF-lac)205(U169)*, *supE44*) (30) was used for cloning, protein production, and *in vivo* assays. *E. coli* strain KA13 (genotype as KA12, additionally carrying λ (DE3) [UV5 P*_lac_*-expressed T7 RNA polymerase gene, *imm21*, Δ*nin5*, *Sam7* (*int*^−^)]) (55, 56) was used for overproduction of MtDS using plasmid pKTDS-HN (11). The plasmid pKIMP-UAUC was used in selection experiments (30). It carries the chloramphenicol resistance gene (*cat*) and provides genes tyrA* and *pheC* encoding monofunctional forms of prephenate dehydrogenase (PDH) from *Erwinia herbicola* and prephenate dehydratase (PDT) from *Pseudomonas aeruginosa*, respectively. Plasmid pT7POLTS (31) was used for protein overproduction in strain KA12. It carries *cat*, the p15A origin of replication, and the genes for both the tetracycline repressor (*tetR*) and the P_*tet*_-controlled T7 RNA polymerase fused to a C-terminal SsrA degradation tag. The latter allows for reduction of the uninduced intracellular T7 RNA polymerase concentration by targeting it to the ClpXP protease system, thus alleviating potential toxic effects of genes due to leaky expression during the growth phase (31).

Plasmids pKECMB-W (57), pKSS (58), pKTCTET-0 (31), pKSS-TM4 (31), pKTNTET (31), pKTCMM-H (11), and pHS10-3p5 (38) were described previously. Plasmid pMG248 (3681 bp) contains a 5′-truncated nonfunctional *aroQ_δ_* gene fragment. It was assembled by ligating the 3462-bp BglII-BlpI fragment from pMG210 (16) with a 219-bp BglII/BlpI-digested PCR product generated with primers 300-TEMP (5′-TAAGATGCTCAGCGAGATCGACCGGCTAGA, restriction sites underlined) and 301-TEMP (5′-TAAAGATCTGTGACCGAGGCGGCCACGGCCCAAT) using plasmid pMG242 (31) as template.^3^

### Acceptor plasmids for aroQ_δ_ gene libraries

The initial acceptor vector pKTNTET-0 for the first *aroQ_δ_* gene libraries codes for an N-terminal Met-hexahistidine tag linked by Ser-Ser-Gly to the Met^5^-Ser^39^ fragment of MtCM. pKTNTET-0 was constructed from the 2703-bp and 1052-bp fragments obtained from a quadruple restriction endonuclease digestion of pKTCTET-0 (31) with enzymes NdeI, BlpI, NheI, and AscI and two correspondingly digested PCR products generated with oligonucleotides 383-dNhe-S (5′-GCTAATCTAGAAGCACGCCATAGTGACTG) and 384-Hind-N (5′-CCTAAGCTCAGCATAAGCTTCCGCAGCCACTAGTCATTATTAGTGGTGGTGGT) on pKTCTET-0 (PCR product cut with XbaI and BlpI, 209 bp) and oligonucleotides 352-CTLIB-S (5′-CCTGTTCATATGCACCATCATCATCACCACTCTT) and 385-NheAsc-N (5′-GGTAAGGCGCGCCCGCTAGCCATCCGGGCCTTGCCGAT) on pKSS-TM4 (PCR product digested with NdeI and AscI, 170 bp). A four-fragment ligation yielded the desired 4134 bp pKTNTET-0, thereby removing the unwanted NheI restriction site in the stuffer fragment.

Acceptor vectors for L1.13, L4.4, and L4.7 libraries were constructed by ligating an NheI/HindIII-digested 1261-bp stuffer fragment from pKTNTET-0 with the correspondingly digested 2873-bp fragment of plasmids pKTNTET-L1.13, pKTNTET-L4.4, and pKTNTET-L4.7, containing the genes of the selected variants L1-13, L4-4, and L4-7 (Table S2), respectively. This yielded the 4134-bp acceptor vectors pKTNTET-0-L1.13, pKTNTET-0-L4.4, and pKTNTET-0-L4.7.

To avoid a second AscI site in the acceptor vectors for the L3.6, L3.7, and L3.8 libraries, the AscI/HindIII-digested 1253-bp stuffer fragment from pKTNTET-0 was ligated with the correspondingly digested 2833-bp fragment from pKTNTET-L3.6, pKTNTET-L3.7, and pKTNTET-L3.8, containing the genes of the selected variants L3.6, L3.7, and L3.8 (Table S2), respectively, yielding the 4086-bp acceptor vectors pKTNTET-0-L3.6, pKTNTET-0-L3.7, and pKTNTET-0-L3.8, respectively.

The acceptor vector pKTNTET-dHis-0 for the untagged version of the *aroQ_δ_* genes was constructed by cloning the PCR fragment generated with oligonucleotides 131-TERM (5′-CCCTCAAGACCCGTTTAGA) and 533-pKTNTET-dHis (5′-AGATATACATATGCTCGAGTCCCAACCTGTCC), using pKTNTET-0 as the template. The PCR product was restriction-digested with NdeI and SpeI, and the resulting 1378-bp fragment was ligated with the 2726-bp fragment of the accordingly digested pKTNTET-0. The resulting 4104-bp pKTNTET-dHis-0 vector was sequenced using oligonucleotide 60-T7Pro (5′-TAATACGACTCACTATAGGG).

The acceptor vector pKTNTET-RepA for the LS4RepA and LS4RepA2 libraries, which encoded the RepA degradation tag MNQSFISDILYADIES (59–61), was based on pKTNTET-0 as backbone and PCR template. A first PCR product for overlap extension was generated using primers 131-TERM and 538-pKTNTET-Fw-RepA (5′-TTAGCGATATTCTGTATGCGGATATTGAATCCCTCGAGTCCCAACCTGT), which contained the XhoI site but excluded the Met codon following the RepA sequence. A second PCR product was obtained using primers 372-TetPro (5′-AGCTCTAATGCGCTGTTAATCACT) and 539-pKTNTet-Rev-RepA (5′-ATACAGAATATCGCTAATAAAGCTCTGGTTCATATGTATATCTCCTTC) providing an NdeI site. The resulting 257-bp and 1479-bp PCR products that had a 17-bp overlapping sequence were assembled using the external 372-TetPro and 131-TERM primers to give a 1719-bp fragment. After digestion with SpeI and NdeI, the resulting 1423-bp fragment was ligated with the correspondingly digested 2726-bp fragment from pKTNTET-0 to obtain vector pKTNTET-RepA (4149 bp).

### Cassette mutagenesis libraries and selection

Cassette mutagenesis for the evolutionary cycle I was performed with oligonucleotides 368-Lp-TRLVfw (5′-ATCGGCAAGGCCCGGATGGCTAGCGGTGGCNNKNNKNNKNNKCATAGTCGGGAGATGAAGGTCATCGAAC) and 386-LpLib-N2 (5′-GGTTAAAGCTTCCGCAGCCACTAGTTATTAGTGACCGAGGCGGCCACGGCCCAAT) on template pMG248 (carrying a 5′-truncated nonfunctional *aroQ_δ_* gene fragment). The NheI/HindIII-digested 148-bp library fragment was ligated to the accordingly cut 2873-bp pKTNTET-0 acceptor fragment.

For cycle II mutagenesis, PCR fragments were generated using 390-CT-LGHrv-2 (5′-GGTTAAAGCTTCCGCAGCCACTAGTTATTANNNNNNNNNTCGACCACGACCAAGACGCAAAAGCAGGATGGCCAGAT), 424-CT-RLGHrv (5′-GGTTAAAGCTTCCGCAGCCACTAGTTATTANNNNNNNNNNNNACCACGACCAAGACGCAAAAGCAGGATGGCCAGAT), or 426-CT7rv (5′-GGTTAAAGCTTCCGCAGCCACTAGTTATTANNNNNNNNNNNNNNNNNNNNNAAGACGCAAAAGCAGGATGGCCAGAT) together with 379-LpconS (5′-GTTCGCTAGCGGAGGTCCACGTCTTGATCATAGTCGGGAGATGAAGGTCATCGAAC) on template pKSS-TM4 (carrying a 3′-truncated *aroQ_δ_* gene). The crude 163-bp PCR products were digested with NheI and HindIII, and the resulting 148-bp fragments were ligated with the accordingly cut 2873-bp pKTNTET-0 acceptor fragment.

The ligation products were transformed into electrocompetent KA12/pKIMP-UAUC cells (30). The suspension of transformed cells was washed three times with 1× M9 salts (6 mg/ml Na_2_HPO_4_, 3 mg/ml KH_2_PO_4_, 1 mg/ml NH_4_Cl, and 0.5 mg/ml NaCl) (53). For cycle I, the cells were spread on M9c minimal medium plates (1.5% agar), which are based on 1× M9 salts and also contain 0.2% (w/v) d-(+)-glucose, 1 mm MgSO_4_, 0.1 mm CaCl_2_, 5 μg/ml thiamine-HCl, 5 μg/ml 4-hydroxybenzoic acid, 5 μg/ml 4-aminobenzoic acid, 1.6 μg/ml 2,3-dihydroxybenzoic acid, 20 μg/ml Trp, 100 μg/ml sodium ampicillin, and 20 μg/ml chloramphenicol, but lack an inducer for gene expression. Library sizes were determined from M9c+Phe+Tyr plates (minimal M9c medium additionally containing 20 μg/ml Tyr and 20 μg/ml Phe). For selection in cycle II, the minimal M9c plates additionally contained 100 μm pFPhe.

### Construction of destabilized Leu^24^ and Leu^31^ MtCM variants

Libraries L1, L2, L3, and L4 were constructed by overlap-extension PCR, using plasmid pHS10-3p5 (which encodes the His-tagged 3p5 variant) as the template (38). The 143-bp 5′ part of the gene was constructed using the forward primer 352-CTLIB-S (5′-CCTTGTTCATATGCACCATCATCATCACCACTCTT) and an individual reverse primer for each library that introduced an AscI restriction site, removed the BsaHI site, and randomized specific codons. The reverse primers 505-L24-N (5′-AAACCTCGGCGCGCCGCTTGACTAACGCAGGATTTCAGCATCMNNACGGTCGATCTCTTCGCGCA, randomizing Leu^24^ via the NNK codon), 506-L31-N (5′-AAACCTCGGCGCGCCGCTTAACMNNAGCGAGGATTTCAGCATCTAGCCGGTCGATCTCTT, randomizing Leu^31^ via NNK), 507-L24-L31-N (5′-AAACCTCGGCGCGCCGCTTAACMNNAGCGAGGATTTCAGCATCMNNACGGTCGATCTCTTCGCGCA, randomizing both Leu^24^ and Leu^31^ via NNK), and 508-L24-L31-RNK-N (5′-AAACCTCGGCGCGCCGCTTAACMNYAGCGAGGATTTCAGCATCMNYACGGTCGATCTCTTCGCGCA, randomizing Leu^24^ and Leu^31^ via RNK, which excludes stop codons) were used for the gene libraries L1, L2, L3, and L4 respectively. For all four libraries, the 202-bp 3′ part of the gene was generated using forward primer 509-AscI-S (5′-AAGCGGCGCGCCGAGGTTTCCAAGGCCATCGG, introducing an AscI site) and reverse primer 510-noBsaHI-N (5′-TCACAGCTTCCGCAGCCACTAGTTATTACATAGCATCCGGACCACGACCAAGAC). The 143-bp and 202-bp PCR fragments were assembled using external primers 352-CTLIB-S and 510-noBsaHI-N, and the resulting 326-bp fragment was restriction-digested with XhoI (coincidentally present in the *aroQ_δ_* gene) and SpeI. The obtained 260-bp fragments from L1, L2, L3, and L4 libraries were ligated with the 2761-bp XhoI-SpeI fragments of pKTNTET-0 yielding the 3021-bp library plasmids.

After transformation of electrocompetent KA12/pKIMP-UAUC and washing twice in 1× M9 salts, the transformants were plated onto relaxed-stringency M9c+Phe+Tet^500ng/ml^ minimal agar (containing M9c medium as described above, with 500 ng/ml tetracycline to induce *aroQ_δ_* gene expression and 20 μg/ml Phe). A small fraction of washed cells were also plated onto nonselective M9c+Phe+Tyr plates for library size estimation. Typically, the *aroQ_δ_* gene of five clones from these plates was sequenced to determine the quality and mutation rates of the library. Clones from M9c+Phe+Tet^500ng/ml^ plates were picked and streaked out onto higher-stringency plates, such as M9c+Tet^80ng/ml^, M9c without additives, and M9c+pFPhe^40μm^ (containing 40 μm toxic pFPhe). Clones not growing after 72 h at 30 °C on these stringent plates were sequenced using 131-TERM.

### Construction of epL1.13, epL4.4, epL4.7, epL3.6, epL3.7, epL3.8, shL1.13, shL4.4, shL4.7, shL3.6, shL3.7, and shL3.8 libraries, and in vivo selection

To obtain the epPCR libraries (epL), two rounds of epPCR were performed on the relatively short *aroQ_δ_* template using the Mutazyme II kit from Stratagene (Agilent Technologies). First, the appropriate DNA of *aroQ_δ_* variants from plasmids pKTNTET-L1.13, pKTNTET-L4.4, pKTNTET-L4.7, pKTNTET-L3.6, pKTNTET-L3.7, and pKTNTET-L3.8 (which encode variants L1.13, L4.4, L4.7, L3.6, L3.7, and L3.6, respectively; Table S2) was amplified using primers 372-TetPro and 131-TERM and 0.5 ng of the template plasmid to yield 591-bp products. The second round of epPCR for the epL3.6, epL3.7, and epL3.8 libraries was done by using 0.5 ng of the 591-bp epPCR product and the 509-AscI-S/131-TERM primer pair to obtain 253-bp products, which were used for cloning after appropriate restriction digestion (see below). For libraries epL1.13, epL4.4, and epL4.7, 0.5 ng of the appropriate 591-bp epPCR products and the 372-TetPro/131-TERM primer pair were used for the second round of epPCR to generate again 591-bp products. In this case, an additional nonmutagenic PCR was performed using primers 509-AscI-S and 131-TERM to obtain the 253-bp products desired for cloning of the epL1.13, epL4.4, and epL4.7 libraries.

DNA shuffling libraries were constructed by shuffling *aroQ_δ_* genes carrying beneficial mutations including the genes from the previously constructed plasmids pHS10-3p5 (*aroQ_δ_* gene encoding variant PHS10-3p5, mutations T52P, V55D, R87P, L88D, G89A, and H90M), pHS08-3p20 (PHS08-3p20, mutation V62I), pHS08-5p12 (PHS08-5p12, mutation G43V), pHS10-ANp10 (PHS10-ANp10, mutation R82Q), and pHS10-2p14 (PHS10-2p14, mutation D75Y) (38). These genes were PCR-amplified using primers 131-TERM and 509-AscI-S to yield 591-bp products. 600 ng of each PCR product were mixed and treated with 1 μg of DNase I until the preferred fragments of ∼50 bp were obtained. These DNA fragments were reassembled by PCR using primers 131-TERM and 509-AscI-S to yield a 253-bp product. If the yield of the reassembly PCR was low, an additional PCR amplification with the same primers was performed.

The 253-bp products of the epPCRs and the reassembly PCR of DNA shuffling for the L1.13, L4.4, and L4.7 libraries were restriction-digested with AcsI and HindIII, and the resulting 188-bp fragments were respectively ligated to the correspondingly digested 2833-bp fragment of acceptor vectors pKTNTET-0-L1.13, pKTNTET-0-L4.4, and pKTNTET-0-L4.7, yielding 3021-bp library plasmids. The 253-bp products of the epPCRs and reassembly PCRs for the L3.6, L3.7, and L3.8 libraries were restriction-digested with AcsI and SpeI, and the resulting 174-bp fragments were ligated to the correspondingly digested 2847-bp fragment of acceptor vectors pKTNTET-0-L3.6, pKTNTET-0-L3.7, and pKTNTET-0-L3.8, respectively, yielding the 3021-bp library plasmids.

The ligations were transformed into electrocompetent KA12/pKIMP-UAUC cells. The cells were washed twice with 1× M9 salts and spread onto the M9c plates (listed by increasing stringency) M9c+Phe+Tet^80ng/ml^, M9c+Phe+Tet^40ng/ml^, M9c+Tet^80ng/ml^, M9c+Tet^40ng/ml^, M9c, M9c+pFPhe^40μm^, and M9c+pFPhe^100μm^ (containing 100 μm pFPhe). Library size and quality were determined from platings on nonselective M9c+Phe+Tyr agar. Colonies still growing at the highest selective conditions were picked and sequenced using oligonucleotide 131-TERM.

### Reversion of destabilizing mutations from evolved inter-subunit destabilized variants

Overlap-extension PCR was used to remove the L24 and L31 mutations from the further-evolved *aroQ_δ_* variants. Fragment PCR I was obtained with oligonucleotides 372-TetPro and 512-x31L-Fw (5′-GACGCCGAAATCCTCGCGTTAGTCAAGCGACGCGCTGAGG) on the appropriate pKTNTET-based template. Fragment PCR II was generated with primers 513-x24L-Rev (5′-CGCGAGGATTTCGGCGTCTAGCCGGTCGATCTCTTCGCGC) and 131-TERM, on the same template. PCR I and PCR II fragments were combined using oligonucleotides 372-TetPro and 131-TERM. The resulting PCR products were digested with XhoI and HindIII to obtain the required 349-bp insert, which was ligated with the 2747-bp fragments from the appropriately digested pKTNTET-0.

### Design and construction of the truncation libraries

The truncation libraries LdNdC and LdNR85X were based on variant re4.7s11 (s11). Each library offered incremental two-residue deletions between the His-tag (including the adjacent Ser-Ser-Gly-Met-Leu-Glu sequence to retain the linker and XhoI site for in-frame cloning) and the active site residue Arg^18^ (Fig. 3). For library LdNdC, the C terminus was also varied by allowing truncations of one residue at a time until Arg^85^ (Fig. 3). This residue, together with Gly^84^, had been shown in previous directed evolution experiments to be conserved and important for catalysis (31). In addition to the random two-residue deletions in the N-terminal region, library LdNR85X members also lacked the last five residues at the C terminus and had codon 85 randomized (Fig. 3).

The LdNdC library of truncated s11 genes was constructed from pKTNTET-re4.7s11 (which encodes the His-tagged s11 variant) as a template and a mixture of several forward and reverse primers. The forward primers 521-MtCMi-N-S8 (5′-GTATGCTCGAGTCCCAACCTGTCCCCGAGATCGACACGC), 522-MtCMi-N-P10 (5′-GTATGCTCGAGCCTGTCCCCGAGATCGACACGCTGC), 523-MtCMi-N-P12 (5′-GTATGCTCGAGCCCGAGATCGACACGCTGCGCGAAG), 524-MtCMi-N-I14 (5′-GTATGCTCGAGATCGACACGCTGCGCGAAGAGATC), 525-MtCMi-N-T16 (5′-GTATGCTCGAGACGCTGCGCGAAGAGATCGAC), and 526-MtCMi-N-R18 (5′-GTATGCTCGAGCGCGAAGAGATCGACCGGCTAG) introduced an XhoI restriction site via the Leu-Glu–encoding sequence directly following the MtCM-native codon for Met^5^ and the respective N-terminal deletions up to residue Ser^8^ (no truncation), Pro^10^, Pro^12^, Ile^14^, Thr^16^, and Arg^18^. The reverse primers 527-MtCMi-C-M90 (5′-CAGCCACTAGTTATTACATAGCATCCGGACCACGACCA), 528-MtCMi-C-A89 (5′-CAGCCACTAGTTATTAAGCATCCGGACCACGACCAAGC), 529-MtCMi-C-D88 (5′-CAGCCACTAGTTATTAATCCGGACCACGACCAAGAC), 530-MtCMi-C-P87 (5′-CAGCCACTAGTTATTACGGACCACGACCAAGACGCAA), 531-MtCMi-C-G86 (5′-CAGCCACTAGTTATTAACCACGACCAAGACGCAAAAGC), and 532-MtCMi-C-R85 (5′-CAGCCACTAGTTATTAACGACCAAGACGCAAAAGC) introduced a SpeI restriction site, two stop codons, and C-terminal truncations back to Met^90^ (no truncation), Ala^89^, Asp^88^, Pro^87^, Gly^86^, and Arg^85^, respectively (Fig. 3).

The PCR product for library LdNdCR85X was constructed using the same template and mixtures of forward primers as described for library LdNdC. The reverse primer was 534-MtCMi-C-R85X (5′-CAGCCACTAGTTATTAMNNACCAAGACGCAAAAGCAG), which introduced a SpeI restriction site and two stop codons after Arg^85^, and randomized Arg^85^ via the NNK codon. The PCR products for libraries LdNdC and LdNdCR85X, which had different sizes depending on the extent of truncation, were digested with XhoI and SpeI and ligated with the 2761-bp fragment of XhoI/SpeI-digested pKTNTET-0.

### Selection of truncated re4.7s11 (s11) variants

The electrocompetent KA12/pKIMP-UAUC cells transformed with the ligation mixtures were plated onto relaxed-stringency M9c+Phe+Tet^500ng/ml^ agar, as described above. Library sizes were calculated from colony growth on nonselective M9c+Phe+Tyr plates, and five clones were sequenced to determine library quality and mutation rate. To confirm a reduced complementation ability of truncated variants, clones from M9c+Phe+Tet^80ng/ml^ plates were picked, inoculated in 100 μl of 1× M9 salts, and drop-spotted onto higher-stringency plates, such as M9c+Phe+Tet^40ng/ml^, M9c+Tet^80ng/ml^, M9c+Tet^40ng/ml^, M9c, and M9c+pFPhe^40μm^ and M9c+pFPhe^100μm^ containing 40 and 100 μm pFPhe, respectively. The clones growing slowest under stringent conditions were picked and sequenced using 131-TERM.

DNA sequencing revealed only one weakly complementing clone (dNdCs1) from library LdNdC that did not grow at higher stringencies (M9c and M9c+pFPhe plates). In contrast, substitutions of Arg^85^ in library LdNdCR85X were severe enough to drastically reduce *in vivo* complementation ability even in the absence of N-terminal lesions. Thus, randomizing Arg^85^ in a variant of s11 lacking the five C-terminal residues yielded variants with reduced activity *in vivo* more frequently than the progressive deletions at the N- and C-terminal regions for library LdNdC members.

### Randomization of truncation libraries based on dNdCs1 and dNdCR85Xs10 and selection experiments

Truncated variants were chosen as templates for the fourth cycle of directed evolution. The genes of variants dNdCs1 (NΔ10, CΔ5; Table S5) and dNdCR85Xs10 (NΔ0, CΔ5, R85M) were mutagenized via two rounds of epPCR using the Mutazyme II kit. The first round involved primers 372-TetPro and 131-TERM and 0.5 ng of plasmids pKTNTET-dNdCs1 or pKTNTET-dNR85Xs10 encoding the His-tagged version of the target variants. 0.5 ng of the respective 546-bp and 576-bp epPCR products were used for a second epPCR round using the forward primer 542-Fw-His (5′-ACTCTTCTGGTATGCTCGAG) and the reverse primers 547-Rv-dNdCs1 (5′-GCAGCCACTAGTTATTAACG for dNdCs1) and 548-Rv-dNR85s10 (5′-GCAGCCACTAGTTATTACAT for dNdCR85Xs10). The XhoI/SpeI-digested 215-bp and 245-bp products were subcloned into the correspondingly digested pKTNTET-0 acceptor vector. The resulting library plasmids carrying the mutagenized dNdCs1 and dNR85s10 genes, respectively, were electroporated into KA12/pKIMP-UAUC. Library size, quality, and mutation rates were determined after plating a fraction of the cells onto nonselective M9c+Phe+Tyr agar plates. *In vivo* selection was performed on plates with M9c+Phe+Tet^80ng/ml^, M9c+Phe+Tet^40ng/ml^, M9c+Tet^80ng/ml^, M9c+Tet^40ng/ml^, M9c+Tet^10ng/ml^, M9c+Tet^5ng/ml^, M9c+Tet^2.5ng/ml^, M9c, and M9c+pFPhe^40μm^ and M9c+pFPhe^100μm^. Clones that grew fastest under stringent conditions were picked and sequenced using oligonucleotide 131-TERM.

### Construction and selection of the RepA-tagged library

To use variant dNdCs4 (NΔ4, CΔ5) as a template for the evolutionary cycle IV, a stepwise evolution protocol was implemented, because this gene already weakly complemented the growth defect of KA12/pKIMP-UAUC. First, an epPCR gene library was generated without the N-terminal His-tag. This was accomplished by mutagenizing the His-tagged dNdCs4 gene (Table S5) via two rounds of epPCR. First, the gene was amplified with primers 372-TetPro and 131-TERM from plasmid pKTNTET-dNdCs4. For the first epPCR, 0.5 ng of this amplified gene served as a template for primers 372-TetPro and 131-TERM using the Mutazyme II kit. Of the resulting 564-bp mutated PCR products, 0.5 ng was used for a second epPCR round using primers 542-Fw-His and 547-Rv-dNdCs1 to give 259-bp fragments. The XhoI/SpeI-digested 233-bp products (without the sequence encoding for His-tag) were subcloned into the correspondingly digested pKTNTET-dHis-0 vector to generate the 2993-bp library plasmid pool, which was electroporated into KA12/pKIMP-UAUC cells.

Library size, quality, and mutation rates were determined from transformants plated onto M9+Phe+Tyr agar and from five sequenced clones. Selection *in vivo* was accomplished by plating onto M9c+Phe+Tet^80ng/ml^, M9c+Phe+Tet^40ng/ml^, M9c+Tet^80ng/ml^, M9c+Tet^40ng/ml^, M9c+Tet^10ng/ml^, M9c+Tet^5ng/ml^, M9c, M9c+pFPhe^40μm^, and M9c+pFPhe^100μm^. After 3 days at 30 °C, well-growing colonies were picked from the most stringent pates and streaked out onto M9c agar. From these streaks, 192 well-growing clones were inoculated into 100 μl of liquid M9c+Phe+Tyr medium in two 96-well plates and incubated overnight at 37 °C. Eight pools of 24 individual clones grown up in 96-well plates were collected and their plasmid DNA was isolated. The target genes were PCR-amplified from each of the eight pools using oligonucleotides 543-Fw-dHis (5′-GAGATATACATATGCTCGAG) and 547-Rv-dNdCs1. The PCR products were gel-purified, pooled to have 800 ng of each fragment, and subjected to DNA shuffling as described above. The DNase I digested products were assembled by PCR with oligonucleotides 543-Fw-dHis and 547-Rv-dNdCs1 and digested with XhoI and SpeI, and the 233-bp fragments were subcloned into the accordingly digested pKTNTET-RepA vector to generate 3009-bp library plasmids. Thereby, the MtCM variants were fused with the N-terminal RepA protein degradation tag (59) with the goal of lowering the intracellular enzyme concentration. After electroporation into KA12/pKIMP-UAUC, the resulting Ls4RepA library transformants were plated on M9c+Phe+Tet^80ng/ml^, M9c+Phe+Tet^40ng/ml^, M9c+Tet^80ng/ml^, M9c+Tet^40ng/ml^, and M9c plates for selection and on M9c+Phe+Tyr plates for determination of library size, quality, and mutation rates.

The best-complementing genes were then again shuffled to create library LS4RepA2, still carrying the RepA tag. For this, two 96-well plates were inoculated as described above with the clones growing on M9c+Tet^80ng/ml^ and M9c+Tet^40ng/ml^ plates. Again, eight pools of individual clones were prepared, plasmid DNA was extracted, and the genes were PCR-amplified using oligonucleotides 544-Fw-RepA (5′-GCGGATATTGAATCCCTCGAG) and 547-Rv-dNdCs1. The PCR products were shuffled, assembled using primers 544-Fw-RepA and 547-Rv-dNdCs1, and cloned into the pKTNTET-RepA vector as described above for the Ls4RepA library. The resulting Ls4RepA2 library was transformed into KA12/pKIMP-UAUC cells and plated onto M9c, M9c+pFPhe^40μm^, and M9c+pFPhe^100μm^ agar plates for selection and on M9c+Phe+Tyr plates for determination of library size, quality, and mutation rates. 150 clones that complemented on high-stringency plates were purified by streaking them out on M9c+pFPhe^100μm^ agar plates. Twenty-three of the best-growing clones were retransformed into KA12/pKIMP-UAUC, tested on M9c+pFPhe^100μm^ agar plates, and sequenced using oligonucleotide 131-TERM.

### Re-elongation of evolved truncated variants

Individual genes for evolved truncated and/or RepA-tagged variants in pKTNTET-based plasmids from libraries LdCdNs1, LdNdCR85Xs10, and Ls4RepA2 were amplified using an appropriate primer pair (summarized in Table S11 and Table S12). The PCR fragments were digested with SpeI/XhoI and the 260-bp inserts ligated with the 2761-bp fragment of the correspondingly digested pKTNTET-0 vector.

### Providing the evolved variants with the WT N terminus

To remove the His-tag and instead provide the evolved variants with the native N terminus, the WT MtCM gene on plasmid pKTCMM-H (11) was replaced by the corresponding fragment of the evolved variant. Restriction digestion with XhoI and SpeI of the pKTNTET-based plasmids that carry the genes of the evolved variants s11 and s4.15 yielded the required 260-bp fragments for ligating with the 4547-bp XhoI-SpeI fragment of pKTCMM-H (11).

### Production and purification of His-tagged proteins

N-terminally His-tagged (pKTNTET-encoded) H_6_-MtCM variants, wherein the four native N-terminal MNLE residues of MtCM were replaced by the sequence MHHHHHHSSG, were produced in KA12/pT7POLTS cells (31). The transformants were grown in 500 ml of LB medium containing 150 μg/ml Na-ampicillin and 30 μg/ml chloramphenicol at 30 °C. Gene expression was induced with 2 μg/ml tetracycline when an *A*_600_ of 0.3–0.5 was reached. The crude lysate was obtained as described before (62) (but without the RNase A and DNase I treatment) and provided with 10 mm imidazole. The sample was loaded onto an equilibrated nickel-nitrilotriacetic acid column containing His-Select Nickel Affinity Gel (Merck, Germany). The MtCM variant was eluted with 250 mm imidazole in 50 mm sodium phosphate buffer, pH 8, containing 0.3 m NaCl and dialyzed against 20 mm potassium phosphate, pH 7.5. Proteins were assessed by SDS-PAGE using the PhastSystem (20% homogeneous gels, GE Healthcare), and their molecular mass (Table S10) was determined by electrospray ionization MS by the Mass Spectrometry Service at the Laboratory of Organic Chemistry, ETH Zurich.

### Production and purification of untagged MtCM variants

The untagged versions of s11 (N-s11; predicted pI = 6.11) and s4.15 (N-s4.15; predicted pI = 6.09), encoded on pKTCMM-H derived plasmids, were produced in *E. coli* strain KA13. The transformants were grown at 37 °C in LB medium containing 150 μg/ml Na-ampicillin. At an *A*_600_ of 0.3–0.6, gene expression was induced by 0.5 mm isopropyl 1-thio-β-d-galactopyranoside and growth continued overnight. After harvesting by centrifugation (5000 rpm for 10 min at 4 °C), the cells were resuspended in 20 mm 1,3-bis[tris(hydroxymethyl)methylamino]propane (BTP) buffer, pH 7.5, and incubated for 1 h on ice with 1 mg/ml lysozyme prior to disruption by sonication. The crude cell lysate was adjusted to 65–70% (w/v) ammonium sulfate and stirred for 1.5–2 h at 4 °C. The precipitate was collected by centrifugation (5880 rpm for 30 min at 4 °C), dissolved in buffer A (20 mm piperazine, pH 9.0), and dialyzed against the same buffer overnight at 4 °C. Dialyzed samples were loaded onto a pre-equilibrated MonoQ column in buffer A. A gradient between 0–30% of buffer B (20 mm piperazine, pH 9, and 1 m NaCl) in buffer A was applied over 50–100 ml at a flow rate of 2 ml/min. The fractions from the MonoQ column were collected, concentrated, and dialyzed against 20 mm MES, pH 5.5 (buffer C). After dialysis, the sample was applied onto a pre-equilibrated MonoS column in buffer C, and the column was washed with 95% buffer C/5% buffer D (20 mm MES, pH 5.5, and 1 m NaCl). The proteins were eluted with a gradient between 5–40% of buffer D in buffer C over 50–100 ml at a flow rate of 2 ml/min. The MonoS column fractions containing the MtCM variants were pooled, concentrated, and loaded onto a Superdex 75 column. Proteins were eluted in 20 mm BTP, pH 7.5, containing 150 mm NaCl. Protein integrity was analyzed by SDS-PAGE and electrospray ionization MS at the Mass Spectrometry Service of the Laboratory of Organic Chemistry, ETH Zurich.

### Production and purification of MtDS

The His-tagged MtDS was produced following the previously established protocol (38) with minor modifications. A single colony of KA13/pKTDS-HN (38) was inoculated into 5 ml of LB containing 150 μg/ml ampicillin (sodium salt) and grown overnight at 30 °C. The resulting pre-culture was used to inoculate M9c minimal medium as described above but containing 150 μg/ml sodium ampicillin and 10 μg/ml each of Trp, Tyr, and Phe. The culture was grown at 30 °C to an *A*_600_ of 0.3–0.5, and protein production was induced with 0.1 mm salicylate. After incubation at 30 °C for 16 h, the cells were harvested and resuspended in BTP++ buffer consisting of 20 mm BTP, pH 7.5, 1 mm tris(2-carboxyethyl)phosphine hydrochloride, 0.2 mm phosphoenolpyruvate, 0.1 mm MnCl_2_, and 150 mm NaCl (11, 63).

The cell pellet was treated with 1 mg/ml lysozyme for 30 min on ice and ruptured by sonication. The insoluble cell debris was removed by centrifugation for 20 min at 13,000 rpm (Sorvall rotor SA600) at 4 °C, and if there was a significant amount of cell pellet left, the sonication was repeated. The crude soluble cell extract was subjected to nickel-nitrilotriacetic acid affinity chromatography (Qiagen, Germany). The bound protein was first washed with BTP++ containing 40 mm imidazole and then with BTP++ containing 40 mm imidazole and 2 m NaCl. MtDS was eluted in BTP++ containing 250 mm imidazole and dialyzed overnight against buffer A (20 mm BTP, pH 7.5, containing 0.1 mm MnCl_2_, 0.2 mm phosphoenolpyruvate, and 1 mm tris(2-carboxyethyl)phosphine hydrochloride)). The sample was further purified by FPLC on a MonoQ HR 10/10 column with buffer A as the running buffer and eluting with a linear gradient of buffer B (buffer A, containing 500 mm NaCl). For storage, a protease inhibitor mixture without EDTA (catalog no. P-8849; Sigma-Aldrich) was added. On average, protein yield was around 1–5 mg/liter of cell culture. The electrophoretic homogeneity of the protein preparations was assessed by SDS–PAGE using the PhastSystem (GE Healthcare).

### Enzyme assays

Steady-state kinetics for AroQ_δ_ variants (with or without the His-tag) were performed at 30 °C in 50 mm potassium phosphate, pH 7.5, by varying chorismate concentrations between 20 and ∼2000 μm. Initial rates were acquired from the initial slopes of absorption decrease curves obtained by measuring chorismate disappearance at 310 nm (ε_310_ = 370 m^−1^ cm^−1^). The protein concentration was determined by a calibrated Bradford assay using BSA as a standard (11). The data were fitted to the Michaelis-Menten equation using KaleidaGraph (Synergy Software, Reading, PA, USA) to derive *k*_cat_ and *K_m_*. *k*_cat_ is calculated per active site. Fig. S6 shows the kinetic data and fitted Michaelis-Menten plots for four representative MtCM variants. Where error ranges are given, mean and standard deviations (σ_n−1_) were calculated from data from at least two independently produced and isolated enzyme preparations. The kinetic characterization of the AroQ_δ_ variants in the presence of MtDS was performed at 274 nm as described before (11) and is detailed in the legend to Table S8.

### CD spectroscopy

Thermal denaturation curves were determined using CD measurements at 222 nm (bandwidth 1 nm) using an Aviv 202 CD spectrometer (Aviv Biomedical, Lakewood, NJ, USA). Approximately 4 μm protein in 20 mm potassium phosphate, pH 7.5, was used in quartz cuvettes with a 0.2-cm path length. After initial equilibration at 10 or 20 °C for 5 min, the sample was heated in 0.2 °C steps with 0.4-min equilibration time and 3-s signal averaging time per data point. The melting was monitored between 10 or 20 °C and 95 °C. The melting temperature (*T*_m_) was calculated from fitting the mean residue ellipticity (Θ) to a sigmoidal curve between 60 and 95 °C, as exemplified in Fig. S7 with representative MtCM variants, using the equation shown in the figure's legend.

### Crystallization, data processing, and structure refinement

Crystallization of N-s4.15 was performed using hanging drop setups at 20 °C and a protein concentration ranging from 5–11 mg/ml (in 20 mm BTP, pH 7.5, and 150 mm NaCl). N-s4.15 crystals grew in conditions containing 0.1 m 2-amino-2-(hydroxymethyl)propane-1,3-diol (Tris-HCl), pH 8.5, 0.2 m trimethylamine N-oxide, and 25% w/v PEG 2000 MME (optimization of JCSG+ crystallization screen, Molecular Dimensions). Diffraction quality crystals were obtained with microseeding. The protein solution was pre-incubated with TSA (from a stock previously synthesized by Dr. Rosalino Pulido as described (64)) by adding a few flakes of the compound 30 min before crystallization setup. Prior to data collection, the crystal was soaked <5 min in mother liquor additionally containing 20% ethylene glycol and flash-cooled in a nitrogen cryo-stream (100 K).

Diffraction data were collected at beamline BM14 at the European Synchrotron Radiation Facility (ESRF, Grenoble, France). Diffraction and refinement data are summarized in Table 2. The data were processed and scaled with *XDS* (65) and merged using the program *AIMLESS* (66) from the *CCP4* program suite (67). The structure was solved by molecular replacement with the program *Phaser* (68), using as a search model the WT MtCM structure (PDB ID: 2W1A, (11)), with TSA ligand, N terminus, C terminus, and H1-H2 loop (Ala^45^-Ser^57^) removed. The structures were refined by alternating rounds of rigid body refinement with *REFMAC5* (69, 70) and model building using *Coot* (71). *PHENIX* (72) was used to generate composite OMIT-maps to validate ambiguous regions for avoiding model bias during model building. Despite the presence of TSA in the crystallization condition, the data contained no electron density that could be attributed to this ligand, and it was therefore not modeled. N-terminal residues prior to Leu^11^ and C-terminal residues Ala^89^ and Met^90^ also lack electron density and were thus not included in the structural model deposited in the Protein Data Bank (73) with PDB ID: 5MPV.

### Preparation of figures

Structure figures were created using PyMOL (version 2.3.1; Schrödinger LLC) with standard alignment parameters. We adhered to the following color scheme: *green*, MtCM in the MtCM-MtDS complex with TSA (PDB ID: 2W1A (11)); *cyan*, MtCM in the MtCM-MtDS complex without TSA (PDB ID: 2W19 (11)); *orange*, MtCM with malate (PDB ID: 2VKL (11)); *yellow*, apo MtCM (PDB ID: 2QBV (41)); and *purple/pink*, autonomous MtCM variant N-s4.15 (PDB ID: 5MPV, this work).

## Data availability

The coordinates and structure factors of the 1.5 Å crystal structure of the autonomous MtCM variant N-s4.15 have been deposited in the Protein Data Bank with the following code: 5MPV. The authors declare that all other data supporting the findings of this study are available within the article and its supporting information.

## Supplementary Material

Supporting Information

## Footnotes

3 M. Gamper, unpublished results.
