# Supplementary material for: Evolving the naturally compromised chorismate mutase from Mycobacterium tuberculosis to top performance
Source: J Biol Chem. 2020 Oct 9;295(51):17514–34. doi: 10.1074/jbc.RA120.014924 (PMC7762937; doi:10.1074/jbc.RA120.014924)
Supplement: Supporting Information [file supp_295_51_17514__index.html]

Evolving the naturally compromised chorismate mutase from Mycobacterium tuberculosis to top performance — Evolving a naturally compromised enzyme — Evolving the naturally compromised chorismate mutase from Mycobacterium tuberculosis to top performance — Evolving a naturally compromised enzyme — Supporting Information 

# Evolving the naturally compromised chorismate mutase from *Mycobacterium tuberculosis* to top performance

## Supporting Information

- Kamarauskaite\_Kast\_JBC\_Supporting\_Info - This file contains additional figures for illustration of the findings and supplementary tables with additional data gathered from individual directed evolution rounds.
