# Supplementary material for: Evolving the naturally compromised chorismate mutase from Mycobacterium tuberculosis to top performance
Source: J Biol Chem. 2020 Oct 9;295(51):17514–34. doi: 10.1074/jbc.RA120.014924 (PMC7762937; doi:10.1074/jbc.RA120.014924)
Supplement: Supporting Information [file supp_RA120.014924_161689_2_supp_609272_qhw1b2.pdf]

Supporting Information for

Evolving the Naturally Compromised Chorismate Mutase from *Mycobacterium tuberculosis* to Top Performance

Jūratė Fahrig-Kamarauskaitė[1], Kathrin Würth-Roderer[1], Helen V. Thorbjørnsrud[2], Susanne Mailand[1], Ute Krengel[2*], Peter Kast[1*]

[1]Laboratory of Organic Chemistry, ETH Zurich, Vladimir-Prelog-Weg 3, CH-8093 Zurich, Switzerland
[2]Department of Chemistry, University of Oslo, P.O. Box 1033 Blindern, NO-0315 Oslo, Norway

[*]Corresponding authors: E-mails: ute.krengel@kjemi.uio.no; kast@org.chem.ethz.ch

This PDF file includes: Pages

I. Figures S1 to S7 S-2 to S-8
II. Tables S1 to S12 S-9 to S-19

I. Supporting Figures

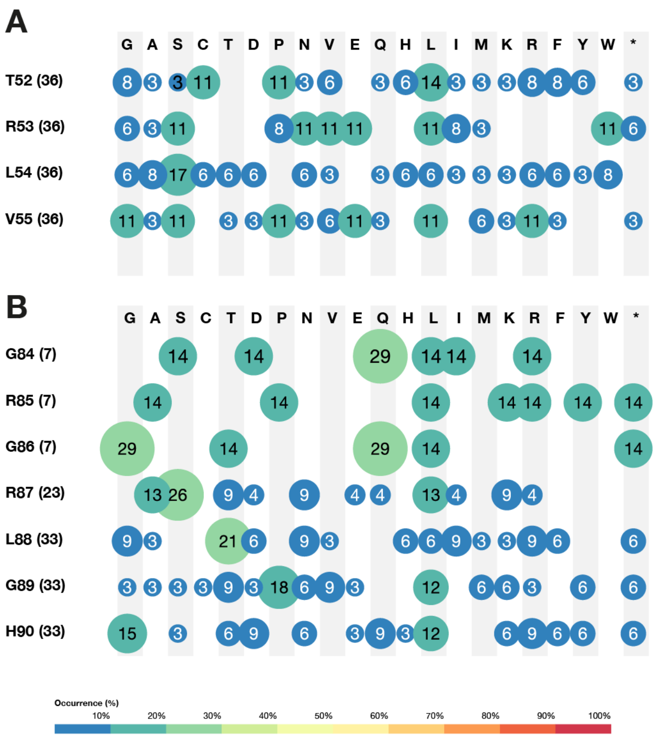

Figure S1. Sequence patterns in randomly picked non-selective clones from the cassette mutagenesis libraries. *A*, amino acid distribution derived from 36 TRLV library members (Cycle I of directed evolution) prior to selection. *B*, for Cycle II, 33 randomly picked pre-selection CT7, CT-LGH, and CT-RLGH library clones were sequenced. The size and color (spectrum shown below panel *B*) of the circles correlate with the occurrence of individual amino acids (in one-letter code; an asterisk represents a stop codon). The circles contain the percentage of a particular residue found at each randomized position, whereas the absolute number of codons sampled in this compilation is given on the left in parentheses, next to the randomized wild-type residue and position. Prior to selection there was no significant sequence bias among clones, neither at the nucleotide (not shown) nor at the amino acid level (see the data in the figure).

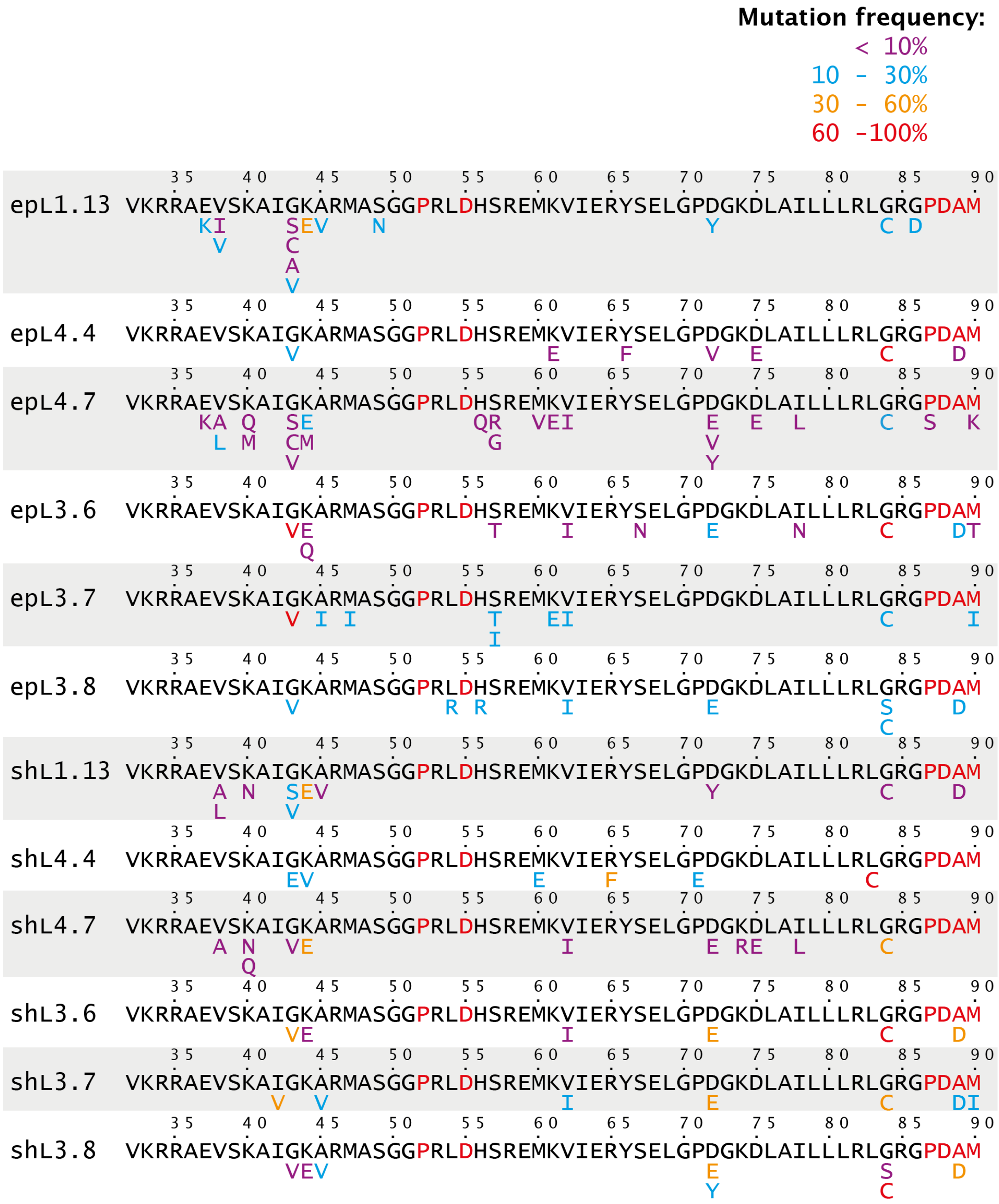

Figure S2. Frequency of mutations in selected clones from inter-subunit destabilization libraries. Sequencing results of selected clones from epPCR (ep) and DNA shuffling (sh) libraries using destabilized variants L1.13, L3.6, L3.7, L3.8, L4.4, or L4.7 as templates are shown. A total of 26, 21, 24, 12, 9, and 6 clones from epPCR libraries epL1.13, epL4.4, epL4.7, epL3.6, epL3.7, and epL3.8, respectively, and 23, 9, 26, 11, 10, and 11 from sh libraries shL1.13, shL4.4, shL4.7, shL3.6, shL3.7, and shL3.8 with genuine sequences were analyzed (cloning artifacts, such as contaminating wild-type sequences, were omitted). The frequency of appearance of a specific mutation is color coded as purple (<10 %), blue (10-30 %), orange (30-60 %), and red (60-100 %). Only the sequence region C-terminal of Leu31, which was subjected to mutagenesis, is shown. Mutations that were already present in the parental 3p5 sequence are highlighted in red for each library template. Clones analyzed prior to selection did not show a bias, neither at the nucleotide nor at the amino acid level (data not shown).

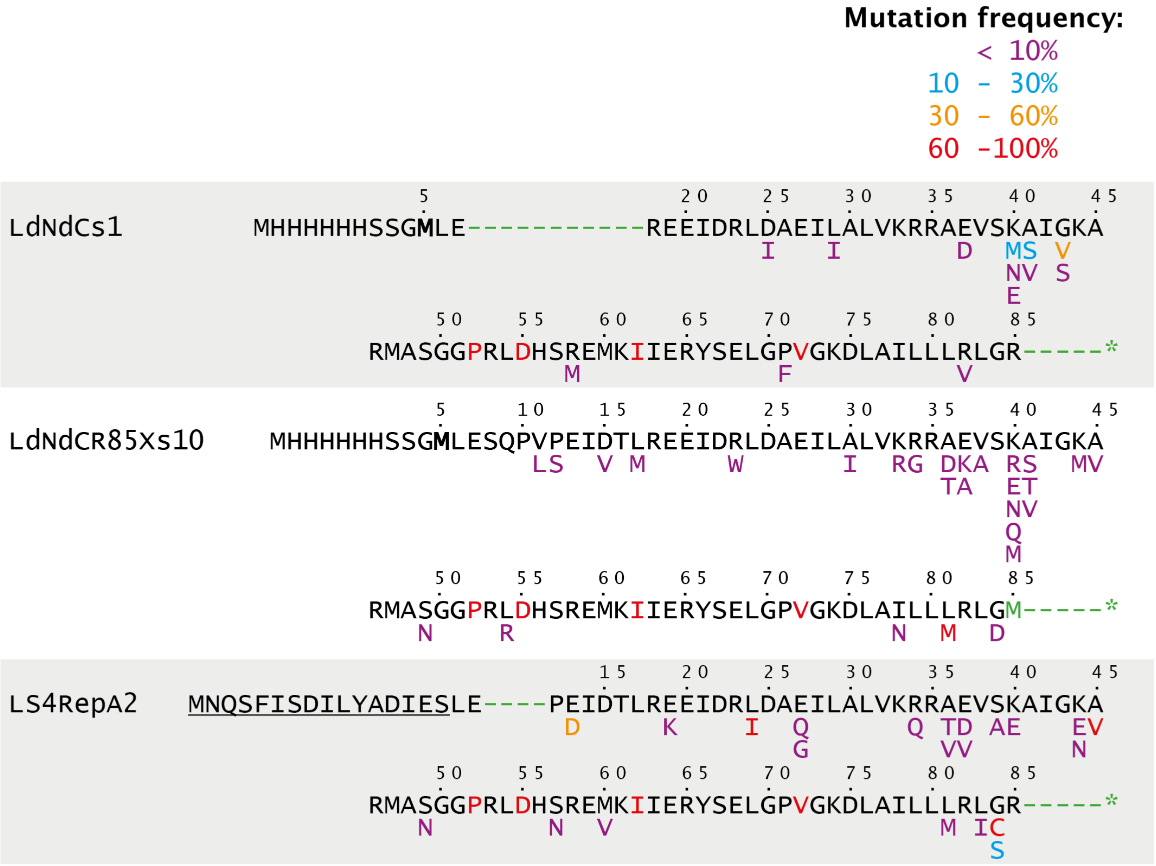

Figure S3. Frequency of mutations in selected clones from truncation libraries. Sequencing results of selected clones from epPCR libraries based on truncated variants dNdCs1 (Library LdNdCs1, N and C-terminal deletions indicated by green dashes) and dNdCR85Xs10 (Library LdNdCR85Xs10, C-terminal deletion and R85M mutation indicated in green), as well as from the dNdCs4-derived library Ls4RepA2 (after epPCR and two rounds of DNA shuffling; RepA tag underlined, N and C-terminal deletions indicated by green dashes). A total of 23, 21, and 16 clones from LdCdNs1, LdNdCR85Xs10, and Ls4RepA2 libraries with genuine sequences were compiled, respectively, omitting artifacts, such as variants that lost their RepA tag due to insertions (> 30%) or deletions (14%). The frequency of appearance of a specific mutation is color coded as purple (<10%), blue (10-30%), orange (30-60%), and red (60-100%). Mutations that were already present in the parent mutant are highlighted in red for each library template. No bias was apparent in clones prior to selection, neither at the nucleotide nor at the amino acid level (data not shown).

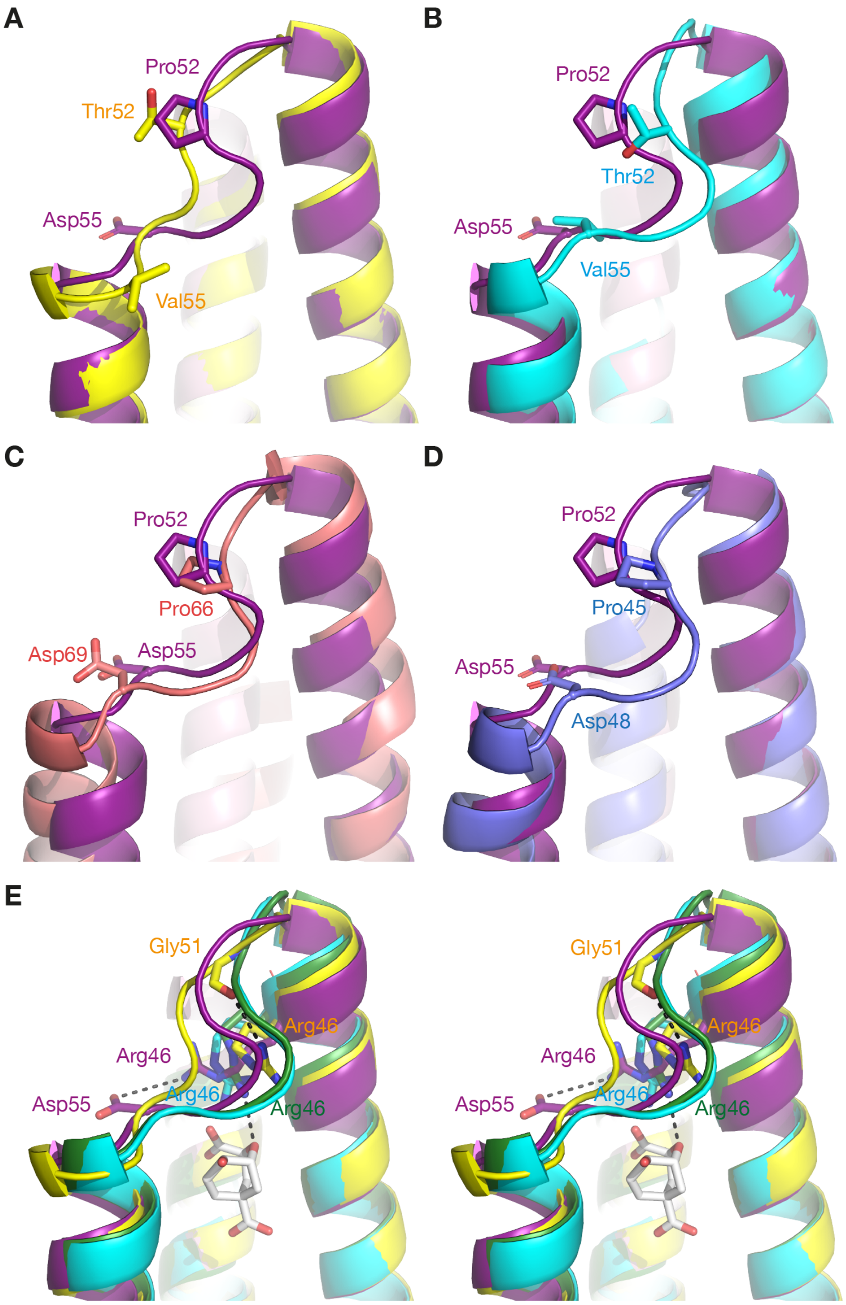

Figure S4. Superimposition of H1-H2 loops. The H1-H2 loop region of N-s4.15 (purple; PDB ID: 5MPV, this work) is compared with corresponding regions of the following MtCM structures: *A*, apo MtCM (yellow; PDB ID: 2QBV, (41)); *B*, MtDS-bound apo MtCM (cyan; PDB ID: 2W19, (11)); *C*, apo *MtCM (red; PDB ID: 2FP1, (20)), and *D*, EcCM (slate blue; PDB ID: 1ECM, (13)). *E*, stereo image illustrating the conformation of the catalytic Arg46 in N-s4.15 (purple), apo MtCM (yellow; PDB ID: 2QBV, (41)), MtDS-bound apo MtCM (cyan; PDB ID: 2W19, (11)), and MtDS-bound MtCM (green; PDB ID: 2W1A, (11)) in complex with TSA (white sticks). Interactions between Arg46 and *(i)* Asp55 (in N-s4.15), *(ii)* the backbone oxygen of Gly51 (in apo MtCM), and *(iii)* the ether oxygen of the TSA (in MtDS-bound MtCM) are indicated by black dashed lines.

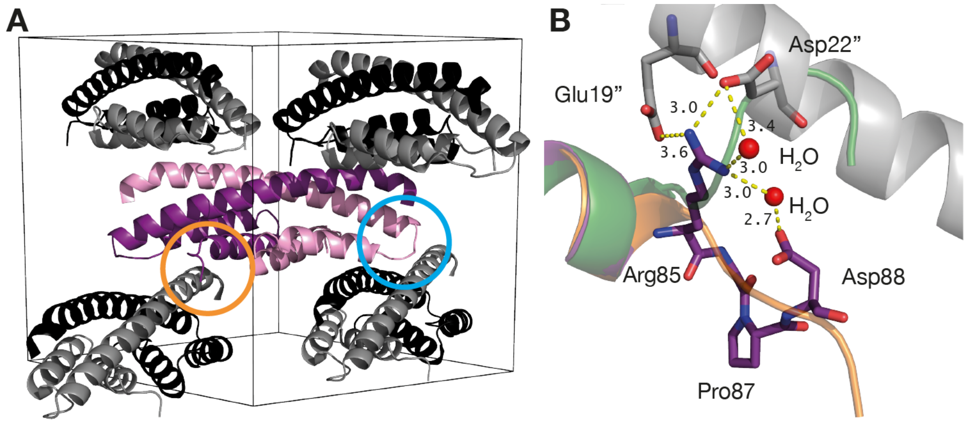

Figure S5. Crystal packing and C-terminal interactions of N-s4.15. *A*, the top-evolved MtCM variant N-s4.15 exhibits extensive crystal contacts at the C-terminus and the catalytically important H1-H2 loop (indicated by orange and blue circles, respectively). The $P6_4$ crystal contains one CM protomer per asymmetric unit. The CM dimer (purple/pink) is generated by 2-fold crystallographic symmetry. Other symmetry-related molecules are colored black/grey and the unit cell is depicted as a black box from the perspective of the γ (120°) angle. *B*, superimpositions of the C-terminal regions of N-s4.15 (purple), malate-bound MtCM (orange; PDB ID: 2VKL, (11)), and MtDS-bound activated MtCM (green; PDB ID: 2W1A, (11)). Polar interactions between Arg85 and side chains of a crystallographic partner (in grey, denoted by ") are indicated by dashed yellow lines.

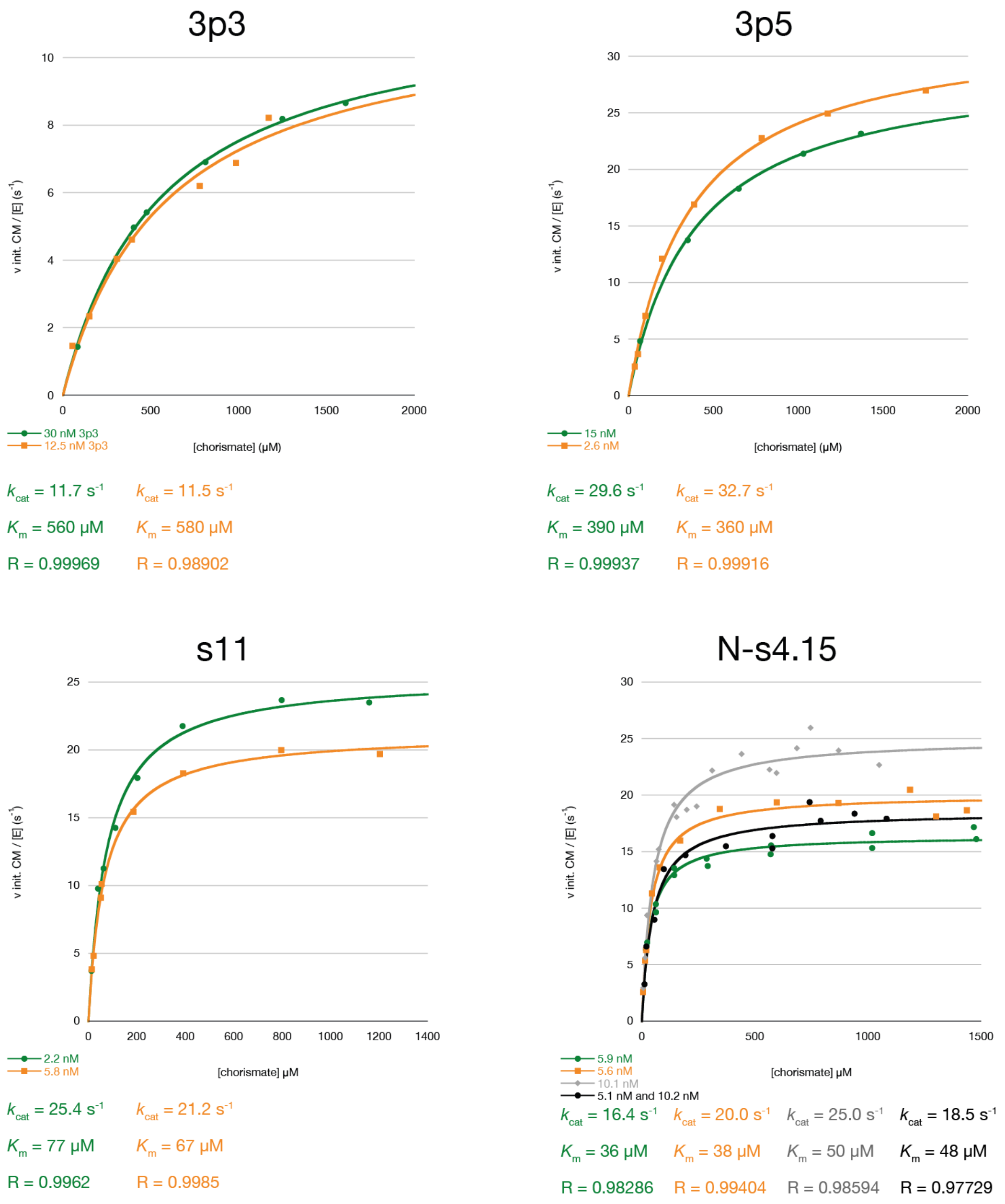

Figure S6. Kinetic data for representative MtCM variants from the evolutionary trajectory. Shown are sets of background-corrected initial velocity data of the depletion of chorismate at 310 nm at 30 °C in 50 mM potassium phosphate, pH 7.5, normalized by the enzyme concentration ($v_{\text{init. CM}}$/[E]; colored dots), for a range of chorismate concentrations ([S]). The plots represent the CM activity of independently produced proteins, assayed at the enzyme concentrations indicated, for the four representative MtCM variants 3p3, 3p5, s11, and N-s4.15. The curves (R = correlation coefficient) illustrate the fitting of the individual data sets to the Michaelis-Menten equation (see below) to derive the catalytic parameters employed for data averaging in Table 1:

$$\frac{v_{\text{init.}}}{[\text{E}]} = \frac{k_{\text{cat}} \cdot [\text{S}]}{K_{\text{m}} + [\text{S}]}$$

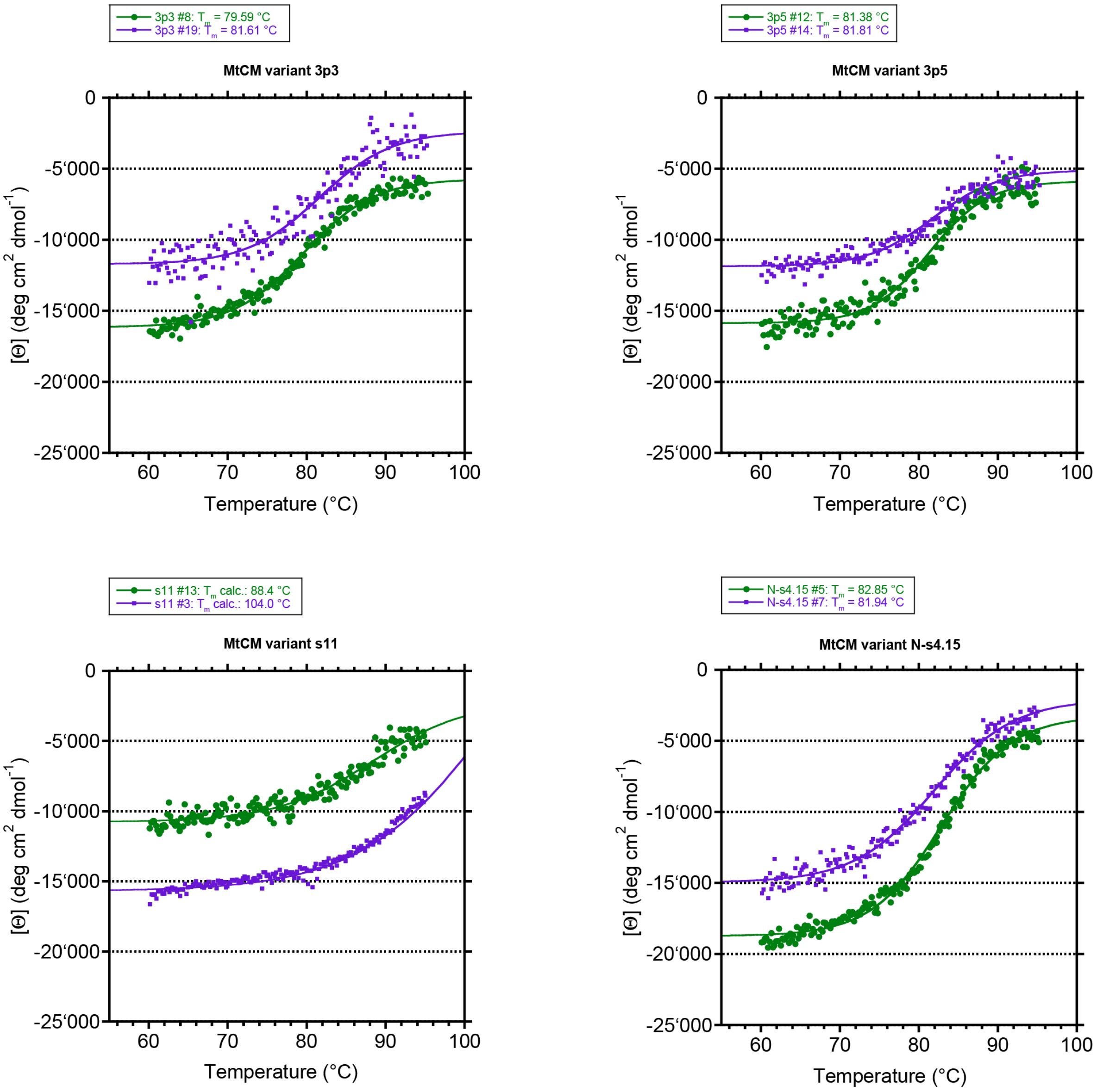

Figure S7. Data sets for melting temperature determination by circular dichroism spectroscopy of representative MtCM variants from the evolutionary trajectory. Independently produced and purified batches of MtCM variants 3p3, 3p5, s11, and N-s4.15 (each set differentiated by batch numbers and colors in the panels) were subjected to thermal denaturation while recording the CD signal at 222 nm. Fitting the mean residue ellipticity (Θ) between 60 and 95 °C to the equation shown below, the melting temperature (T_m) was derived as the inflection point of the sigmoidal curve (80).

$$[\theta] = a + b/(1 + e^{(T - T_m) \cdot c})$$

II. Supporting Tables

Table S1. **Primary sequence and kinetic properties of characterized MtCM variants from the cassette mutagenesis libraries**

His_6-tagged protein	Source library	Mutations[a]	k_{cat}[b] (s^{-1})	K_m[b] (μM)	k_{cat}/K_m[b] ×10^4 (M^{-1} s^{-1})
PHS08-3p3	^{52}TRLV^{55}	T52P/V55D	12	570	2.1
MtCM-T52P	–	T52P	3.5	620	0.56
MtCM-V55D	–	V55D	ND	>700	1.1
PHS10-6p8	PD/CT7	PD/CRGRLNV	14	550	2.5
PHS11-5p3	PD/CT7	PD/SRGESA	ND	>1,300	1.2
PHS11-6p2	PD/CT7	PD/GRGPTHK	18	770	2.3
PHS11-1p4	PD/LGH	PD/PDV	24	560	4.3
PHS10-CGp41	PD/LGH	PD/DEQ	ND	>700	3.9
PHS11-2p2	PD/LGH	PD/DAD	26	670	3.9
PHS10-1p1	PD/LGH	PD/IEG	ND	>700	2.6
PHS10-3p5	PD/RLGH	PD/PDAM	31	380	8.3
PHS11-3p1	PD/RLGH	PD/PDTP	27	560	4.8
PHS11-4p8	PD/RLGH	PD/PDER	ND	>700	4.3
PHS10-CSp49	PD/RLGH	PD/PDML	ND	>800	4.1
PHS10-4p10	PD/RLGH	PD/PGPA	18	600	3.0

[a] PD indicates replacements T52P and V55D; PDAM indicates replacements R87P, L88D, G89A, H90M.
[b] CM activity was determined at 30 °C in 50 mM potassium phosphate buffer, pH 7.5, containing 0.1 mg/mL BSA. Disappearance of chorismate was monitored at 310 nm (ε_{310} = 370 M^{-1} cm^{-1}). Kinetic parameters were derived by fitting initial velocities of chorismate consumption at ≥5 different chorismate concentrations between 20 and ~2000 μM to the Michaelis-Menten equation; ND: not determined, since substrate saturation could not be achieved using up to 2 mM chorismate. Measuring at higher than 2 mM substrate concentrations was not possible due to the high chorismate absorption of the substrate at 310 nm and the relatively high background reaction.

Table S2. Effects of mutations on the kinetic parameters and melting temperature of destabilized 3p5 variants

His_6-tagged protein	Destabilizing mutations	Parental mutations[a]	k_{cat}[b] (s^{-1})	K_m[b] (μM)	k_{cat}/K_m[b] $\times 10^4$ (M^{-1} s^{-1})	T_m[c] (°C)
3p5	–	PD/PDAM	31 ± 2	380 ± 20	8.3 ± 0.8	82 ± 0
L1.13	L24E	PD/PDAM	10	880	1.0	ND
L4.4	L24G, L31R	PD/PDAM	ND	>1,800	0.05	ND
L3.7	L24R, L31R	PD/PDAM	ND	>1,700	0.2	34
L3.8	L24Q, L31G	PD/PDAM	16	1,300	1.1	39
L3.6	L24W, L31T	PD/PDAM	21	430	5.0	49
L4.7	L24T, L31S	PD/PDAM	28	550	5.0	ND

[a, b] See the corresponding footnotes of Table S1.

[c] The melting temperature was determined by CD spectroscopy following the procedure described in Materials and Methods; ND: not determined.

Table S3. **Kinetic parameters of inter-subunit destabilized MtCM variants following Cycle III of directed evolution**

His_6-tagged protein	Destabilizing mutations	Parental mutations[a]	New mutations	k_{cat}[b] (s^{-1})	K_m[b] (μM)	k_{cat}/K_m[b] $\times 10^4$ (M^{-1} s^{-1})
L1.13	L24E	PD/PDAM	–	10	880	1.2
ep1.13s1	L24E	PD/PDAM	G43V	3.6	42	8.4
ep1.13s4	L24E	PD/PDAM	K44E	2.5	24	10
sh1.13s2	L24E	PD/PDAM	V38L	ND	>1,500	1.5
sh1.13s13	L24E	PD/PDAM	G43S	ND	>3,000	0.4
1-3	L24E	PD/PDM	A45V, D72Y, A89D	27	500	5.4
1-14	L24E	PD/PDAM	E37K, G84C	17	170	10
2-2	L24E	PD/PDAM	V38L	ND	>1,600	2.1
2-20	L24E	PD/PDAM	G43A	ND	>1,300	1.7
L4.4	L24G, L31R	PD/PDAM	–	ND	>1,900	0.05
ep4.4s1	L24G, L31R	PD/PDAM	G84C	22	670	3.3
ep4.4s8	L24G, L31R	PD/PDAM	D75E	ND	>1,400	3.4
3.4-s1	L24G, L31R	PD/PDAM	Y66F, G84C	24	940	2.5
3-8	L24G, L31R	PD/PDAM	D72E, G84C	21	1,700	1.3
4-s2	L24G, L31R	PD/PDM	G84C, A89D	35	660	5.3
4-e3	L24G, L31R	PD/PDM	G43V, A89D	30	1,200	2.5
L4.7	L24T, L31S	PD/PDAM	–	28	550	5.0
ep4.7s11	L24T, L31S	PD/PDAM	V62I, D72V	47	180	27
ep4.7s13	L24T, L31S	PD/PDAM	D72E, G84C	22	248	8.9
sh4.7s2	L24T, L31S	PD/PDAM	K40Q	ND	>2,300	1.5
sh4.7s3	L24T, L31S	PD/PDAM	V62I	18	209	8.8
5-8	L24T, L31S	PD/PDAM	K44E, G84C	16	390	4.1
5-15	L24T, L31S	PD/PDAM	K44M, D72E	ND	>1,600	2.3
L3.6	L24W, L31T	PD/PDAM	–	21	430	4.8
sh3.6s22	L24W, L31T	PD/PDAM	K44E, D72E, G84C	8.5	190	4.5
ep3.6s23	L24W, L31T	PD/PDAM	G43V, S67N, I78N	2.5	93	2.7
sh3.6s25	L24W, L31T	PD/PDM	V62I, G84C, A89D	2.5	57	4.4
ep3.6s25	L24W, L31T	PD/PDA	G43V, M90T	1.2	30	4.1
ep3.6s26	L24W, L31T	PD/PDAM	K44Q, V62I	3.5	120	2.9
ep3.6s27	L24W, L31T	PD/PDAM	S57T, G84C	2.3	380	0.6
L3.7	L24R, L31R	PD/PDAM	–	ND	>1,700	0.2
ep3.7s6	L24R, L31R	PD/PDA	V62I, G84C, M90I	0.002	140	0.001
sh3.7s11	L24R, L31R	PD/PDAM	A45V, D72E, G84C	13	220	6.1
L3.8	L24Q, L31G	PD/PDAM	–	ND	>1,300	1.1
sh3.8s6	L24Q, L31G	PD/PDAM	A45V, D72E, G84C	1.1	54	2.1
sh3.8s8	L24Q, L31G	PD/PDM	G43V, A89D	0.9	40	2.3
sh3.8s11	L24Q, L31G	PD/PDAM	A45V, D72Y, G84S	2.6	59	4.4
sh3.8s12	L24Q, L31G	PD/PDM	K44E, D72Y, A89D	5.1	270	1.9

[a, b] See the corresponding footnotes of Table S1.

Table S4. Kinetic parameters of Cycle III-evolved inter-subunit destabilized MtCM variants after removing the destabilizing mutations

His_6-tagged protein	Destabilizing mutations	Parental mutations[a]	New mutations	k_{cat}[b] (s^{-1})	K_m[b] (μM)	k_{cat}/K_m[b] $\times 10^4$ (M^{-1} s^{-1})
re1.13s1	–	PD/PDAM	G43V	6.8	48	14
re1-3	–	PD/PDM	A45V, D72Y, A89D	29	180	16
re1-14	–	PD/PDAM	E37K, G84C	25	250	9.8
re4.4s1	–	PD/PDAM	G84C	23	470	4.9
re4.4s8	–	PD/PDAM	D75E	31	460	6.8
re3.4-s1	–	PD/PDAM	Y66F, G84C	15	340	4.4
re4-s2	–	PD/PDM	G84C, A89D	11	140	8.1
re4-e3	–	PD/PDM	G43V, A89D	6.9	230	3.0
re4.7s11	–	PD/PDAM	V62I, D72V	23	72	33
re4.7s13	–	PD/PDAM	D72E, G84C	20	280	6.9
re4.7s3	–	PD/PDAM	V62I	65	440	15
re3.6s22	–	PD/PDAM	K44E, D72E, G84C	14	160	8.8
re3.6s23	–	PD/PDAM	G43V, S67N, I78N	7.6	52	15
re3.6s25	–	PD/PDM	V62I, G84C, A89D	3.4	37	9.2
re3.6s25	–	PD/PDA	G43V, M90T	5.6	24	23
re3.6s26	–	PD/PDAM	K44Q, V62I	21	120	17
re3.6s27	–	PD/PDAM	S57T, G84C	9.7	130	7.6
re3.7s6	–	PD/PDA	V62I, G84C, M90I	5.7	46	12
re3.7s11	–	PD/PDAM	A45V, D72E, G84C	6.5	70	9.3
re3.8s6	–	PD/PDAM	A45V, D72E, G84C	4.3	100	4.3
re3.8s8	–	PD/PDM	G43V, A89D	3.4	34	10
re3.8s11	–	PD/PDAM	A45V, D72Y, G84S	13	91	14
re3.8s12	–	PD/PDM	K44E, D72Y, A89D	21	140	15

[a, b] See the corresponding footnotes of Table S1.

Table S5. The effects of truncations on the kinetic parameters and the melting temperature of truncated re4.7s11 variants

His_6-tagged protein[a]	N-terminus[b]	C-terminus[c]	k_{cat} (s^{-1}	K_m[d] (μM)	k_{cat}/K_m[d] $\times10^4$ (M^{-1} s^{-1})	T_m[e] (°C)
re4.7s11 (= s11)	$MH_6SSGMLESQPVPEIDTLR^{18}$	$^{85}RGPDAM^{90}$	23	72	33	>88
dNdCR85Xu15	$MH_6SSGMLE$----------R^{18}	^{85}------^{90}	ND	ND	0.002	77
dNdCs1	$MH_6SSGMLE$----------R^{18}	^{85}R-----^{90}	ND	ND	0.01	74
dNdCs4	$MH_6SSGMLE$----$PEIDTLR^{18}$	^{85}R-----^{90}	ND	ND	0.03	86
dNdCs3	$MH_6SSGMLE$--$PVPEIDTLR^{18}$	^{85}R-----^{90}	ND	>1,300	0.4	nd
dNdCs2	$MH_6SSGMLESQPVPEIDTLR^{18}$	^{85}R-----^{90}	ND	>3,700	0.3	nd
dNdCR85Xs5	$MH_6SSGMLESQPVPEIDTLR^{18}$	^{85}L-----^{90}	ND	>1,000	0.1	>90
dNdCR85Xs10	$MH_6SSGMLESQPVPEIDTLR^{18}$	^{85}M-----^{90}	ND	>3,600	0.3	>90
dNdCR85Xs48	$MH_6SSGMLESQPVPEIDTLR^{18}$	^{85}K-----^{90}	ND	>2,900	0.2	nd

[a] All truncated variants are descendants of re4.7s11.
[b] The N-terminal region up to position 18 is represented.
[c] The C-terminal region between position 85 and position 90 is represented.
[d] See footnote [b] of Table S1.
[e] See footnote [c] of Table S2.

Table S6. Kinetic parameters of truncated destabilized MtCM variants following Cycle IV of directed evolution

His_6-tagged protein	Destabilizing truncations and mutations[a]	Parental mutations[a]	New mutations	k_{cat}[b] (s^{-1})	K_m[b] (μM)	k_{cat}/K_m[b] ×10^4 (M^{-1} s^{-1})
dNdCR85X-s10	CΔ5, R85M	PD/V62I, D72V	–	ND	>3,600	0.3
6p13	CΔ5, R85M	PD/V62I, D72V	A36T	ND	>1,500	0.2
4p6-14	CΔ5, R85M	PD/V62I, D72V	K33R	ND	>1,600	0.4
4p6-15	CΔ5, R85M	PD/V62I, D72V	A30V	ND	>3,500	0.3
1p11	CΔ5, R85M	PD/V62I, D72V	K40M, L81M	4.3	420	1.0
2p13	CΔ5, R85M	PD/V62I, D72V	P12S, E37A, L81M	12	230	5.4
3p15	CΔ5, R85M	PD/V62I, D72V	E37K, L81M	3.7	180	2.0
5p12	CΔ5, R85M	PD/V62I, D72V	K40N, I78N	ND	ND	0.2
6p11	CΔ5, R85M	PD/V62I, D72V	K40R, A41S, L81M	4	230	1.7
dNdCs1	NΔ10, CΔ5	PD/V62I, D72V	–	ND	ND	0.01
1p5	NΔ10, CΔ5	PD/V62I, D72V	G43V	ND	ND	0.05
5p1	NΔ10, CΔ5	PD/V62I, D72V	A41V	ND	ND	0.02
3p4	NΔ10, CΔ5	PD/V62I, D72V	K40E	ND	>1,500	0.01

[a] CΔ5 and NΔ10 indicates that 5 and 10 residues were deleted at the C and N terminus, respectively. PD represents replacements T52P and V55D.
[b] See footnote [b] of Table S1.

Table S7. **Kinetic parameters of Cycle IV-evolved truncated destabilized MtCM variants after re-elongation**

His_6-tagged protein	Parental mutations[a]	New mutations	k_{cat}[b] (s^{-1})	K_m[b] (µM)	k_{cat}/K_m[b] $\times 10^4$ (M^{-1} s^{-1})
re4.7s11 (= s11)	PD/V62I, D72V/PDAM	–	23 ± 3	72 ± 7	33 ± 5
s10es2.12	PD/V62I, D72V/PDAM	R34G, A45V	21	62	33
s10es2.14	PD/V62I, D72V/PDAM	E37K	35	150	24
s10es3.13	PD/V62I, D72V/PDAM	A41V	6	82	7.3
s10es4	PD/V62I, D72V/PDAM	L17M, V38A	27	92	29
s10es4.15 (= s4.15)	PD/V62I, D72V/PDAM	V11L, D15V, K40Q	14 ± 2	31 ± 7	45 ± 11
s10es5.12	PD/V62I, D72V/PDAM	A36T	27	100	26
s10es5.18	PD/V62I, D72V/PDAM	R23W	20	95	21
s10es6.12	PD/V62I, D72V/PDAM	K33R	52	200	25
s10es6.13	PD/V62I, D72V/PDAM	A36D, S49N, L54R	7.5	59	13
s10es6.14	PD/V62I, D72V/PDAM	K33R	31	110	28
s10es6.15	PD/V62I, D72V/PDAM	A30V	31	89	38
s10es6.17	PD/V62I, D72V/PDAM	K40E	40	230	17
s1es2	PD/V62I, D72V/PDAM	Y66F	43	210	20
s1es2.10	PD/V62I, D72V/PDAM	I21L, G43S	11	67	16
s1es2.3	PD/V62I, D72V/PDAM	L24I, K40N	33	140	23
s1es3	PD/V62I, D72V/PDAM	L29I, K40M	29	160	18
s1es3.1	PD/V62I, D72V/PDAM	I78V	25	120	20
s1es3.2	PD/V62I, D72V/PDAM	K40M	32	85	38
s1es3.4	PD/V62I, D72V/PDAM	I14V, K40E	32	220	15
s1es3.7	PD/V62I, D72V/PDAM	L54F	23	210	11
s1es4	PD/V62I, D72V/PDAM	G43V	2.6	11	24
s1es5	PD/V62I, D72V/PDAM	E37D	27	78	34
s1es5.1	PD/V62I, D72V/PDAM	A41V	35	103	35
s1es6.1	PD/V62I, D72V/PDAM	A41S	28	77	36

(Continued on next page)

(Continued from previous page)

Table S7 (continued). Kinetic parameters of Cycle IV-evolved truncated destabilized MtCM variants after re-elongation

His_6-tagged protein	Parental mutations[a]	New mutations	k_{cat}[b] (s^{-1})	K_m[b] (μM)	k_{cat}/K_m[b] $\times 10^4$ (M^{-1} s^{-1})
s4-repA2-es10	PD/V62I, D72V/PDAM	L24I, A36T, G84C	11	120	9.3
s4-repA2-es101	PD/V62I, D72V/PDAM	L24I, E27G, A45V	16	79	20
s4-repA2-es110	PD/V62I, D72V/PDAM	L24I, R34Q, K40E, L81M, G84S	2.6	300	0.9
s4-repA2-es113	PD/V62I, D72V/PDAM	L24I, G84C	8	33	24
s4-repA2-es116	PD/V62I, D72V/PDAM	E13D, L24I, K44E, G84S	18	150	12
s4-repA2-es16	PD/V62I, D72V/PDAM	L24I, E27G, A45V, G84C	8	48	17
s4-repA2-es22	PD/V62I, D72V/PDAM	E13D, L24I, E37V, K44N, A45V, G84C	15	52	29
s4-repA2-es24	PD/V62I, D72V/PDAM	E27Q, A36V, S57N	17	62	27
s4-repA2-es3	PD/V62I, D72V/PDAM	L24I, A45V, M60V, G84C	10	67	15
s4-repA2-es30	PD/V62I, D72V/PDAM	E19K, L24I	17	74	23
s4-repA2-es31	PD/V62I, D72V/PDAM	E13D, L83I	45	250	18
s4-repA2-es33	PD/V62I, D72V/PDAM	L24I, A45V	23	150	15
s4-repA2-es4	PD/V62I, D72V/PDAM	L24I, E37D	25	150	17
s4-repA2-es60	PD/V62I, D72V/PDAM	E13D, L24I, E27G, S39A, A45V, G84C	15	52	29
s4-repA2-es62	PD/V62I, D72V/PDAM	E13D, E27G, S49N, G84S	9	53	17

[a, b] See the corresponding footnotes of Table S1.

Table S8. **Impact of MtDS on the specific activity of untagged wild-type MtCM and the top-evolved variants**

	Apparent specific activity in µM chorismate/µM MtCM variant[a]		
	MtCM	N-s11	N-s4.15
No MtDS	1.3 ± 0.1	41.3 ± 3.2	52.8 ± 8.6
With MtDS	14.3 ± 1.3	43.5 ± 4.5	48.4 ± 6.4

[a] Apparent specific initial velocities of chorismate consumption were monitored at 274 nm (ε_{274} = 2630 $M^{-1}\cdot cm^{-1}$) using 23 µM chorismate, 5 nM of MtCM variant with or without 50 nM MtDS in 50 mM BTP, pH 7.5, 1 mM TCEP, 0.2 mM PEP, and 0.1 mM $MnCl_2$ at 30 °C. Note that full activation of MtCM required a 200-fold molar excess of MtDS (11). Standard deviations were determined from at least three measurements using two independent preparations of the MtCM variants.

Table S9. **Catalytic efficiencies of natural and artificially evolved CMs**

Chorismate mutase	Source organism	k_{cat} (s^{-1})	K_m (µM)	k_{cat}/K_m ×10^5 (M^{-1} s^{-1})	Literature
H_6-MtCM	*M. tuberculosis H37Rv*	ND	>1,400	0.0094	This study
MtCM	*M. tuberculosis H37Rv*	2.0	1,140	0.018	(11)
mMjCM	*Methanocaldococcus jannaschii* (monomeric)	4.5	222	0.20	(59)
3p3	*M. tuberculosis H37Rv*	12	570	0.21	This study
3p5	*M. tuberculosis H37Rv*	31	380	0.83	This study
MjCM	*Methanocaldococcus jannaschii*	5.7	41	1.4	(56)
*YpCM	*Yersinia pestis*	70	500	1.4	(41)
EcCM	*Escherichia coli*	64	390	1.6	(81)
TtCM	*Thermus thermophilus*	52	290	1.8	(82)
ScCM[a]	*Saccharomyces cerevisiae*	134	600	2.2	(83)
MtCM-MtDS	*M. tuberculosis H37Rv*	8.1	34	2.4	(11)
AtCM2	*Arabidopsis thaliana* (non-allosteric isoform)	38.7	150	2.6	(84)
*MtCM	*M. tuberculosis H37Rv*	50	180	2.8	(16)
s11	*M. tuberculosis H37Rv*	23	72	3.3	This study
N-s4.15	*M. tuberculosis H37Rv*	20	43	4.7	This study
BsCM	*Bacillus subtilis*	41	72	5.7	(81)

[a] Kinetic parameters are taken from the latest ScCM publication by the Braus group that used a revised CM stop assay (83).

Table S10. ESI MS results

MtCM (-variant)	Calculated MW	Experimentally found MW
H_6-MtCM	10,787.42	10,786.8
3p3	10,799.39	10,798.75
3p5	10,750.32	10,749.59
s11	10,748.40	10,747.67
s4.15	10,746.43	10,745.69
N-s11	10,050.72	10,049.40
N-s4.15	10,048.75	10,047.40

Table S11. Primer pairs for elongating individual truncated variant genes

Variant name	Forward primer[a]	Reverse primer[a]
s1-es4	561-dNdCs1-Fw	556-dNdCs4-Rv
s1-es2.10	561-dNdCs1-Fw	556-dNdCs4-Rv
s1-es6.1a	561-dNdCs1-Fw	556-dNdCs4-Rv
s1-es5.1	561-dNdCs1-Fw	556-dNdCs4-Rv
s1-es3.2	561-dNdCs1-Fw	556-dNdCs4-Rv
s1-es3	561-dNdCs1-Fw	556-dNdCs4-Rv
s1-es2.3	561-dNdCs1-Fw	556-dNdCs4-Rv
s1-es3.4	561-dNdCs1-Fw	556-dNdCs4-Rv
s1-es5	561-dNdCs1-Fw	556-dNdCs4-Rv
s1-es3.1	561-dNdCs1-Fw	556-dNdCs4-Rv
s1-es3.7	561-dNdCs1-Fw	556-dNdCs4-Rv
s1-es2	561-dNdCs1-Fw	556-dNdCs4-Rv
s10-es 4	60-T7Pro	556-dNdCs4-Rv
s10-es 2.12	60-T7Pro	556-dNdCs4-Rv
s10-es 2.14	60-T7Pro	556-dNdCs4-Rv
s10-es 5.18	60-T7Pro	556-dNdCs4-Rv
s10-es 4.15	60-T7Pro	556-dNdCs4-Rv
s10-es 3.12	60-T7Pro	562-dNdCs4-Rv
s10-es 3.13	60-T7Pro	556-dNdCs4-Rv
s10-es 5.12	60-T7Pro	563-dNdCs4-Rv
s10-es 6.12	60-T7Pro	556-dNdCs4-Rv
s10-es 6.13	60-T7Pro	556-dNdCs4-Rv
s10-es 6.14	60-T7Pro	556-dNdCs4-Rv
s10-es 6.15	60-T7Pro	556-dNdCs4-Rv
s10-es 6.17	60-T7Pro	556-dNdCs4-Rv
s4-repA2-s 3	554-dNdCs4-Fw	557-dNdCs4-Rv
s4-repA2-s 4	554-dNdCs4-Fw	556-dNdCs4-Rv
s4-repA2-s 10	554-dNdCs4-Fw	557-dNdCs4-Rv
s4-repA2-s 16	554-dNdCs4-Fw	557-dNdCs4-Rv
s4-repA2-s 22	555-dNdCs4-Fw	557-dNdCs4-Rv
s4-repA2-s 24	554-dNdCs4-Fw	556-dNdCs4-Rv
s4-repA2-s 30	554-dNdCs4-Fw	556-dNdCs4-Rv
s4-repA2-s 31	555-dNdCs4-Fw	559-dNdCs4-Rv
s4-repA2-s 33	554-dNdCs4-Fw	556-dNdCs4-Rv
s4-repA2-s 60	555-dNdCs4-Fw	557-dNdCs4-Rv
s4-repA2-s 62	555-dNdCs4-Fw	558-dNdCs4-Rv
s4-repA2-s 101	554-dNdCs4-Fw	556-dNdCs4-Rv
s4-repA2-s 110	554-dNdCs4-Fw	560-dNdCs4-Rv
s4-repA2-s 113	554-dNdCs4-Fw	557-dNdCs4-Rv
s4-repA2-s 116	555-dNdCs4-Fw	558-dNdCs4-Rv

[a] The primer sequences are listed in Table S12.

Table S12. **Primer sequences used for elongating individual truncated variant genes**

Primer	Sequence
554-dNdCs4-Fw	5'-TATGCTCGAGTCCCAACCTGTCCCCGAGATCGACACGCTGC
555-dNdCs4-Fw	5'-TATGCTCGAGTCCCAACCTGTCCCCGATATCGACACGCTGC
556-dNdCs4-Rv	5'-CACTAGTTATTACATAGCATCCGGACCACGACCAAGACGC AAAAGCA
557-dNdCs4-Rv	5'-CACTAGTTATTACATAGCATCCGGACCACGACAAAGACGC AAAAGCA
558-dNdCs4-Rv	5'-CACTAGTTATTACATAGCATCCGGACCACGACTAAGACGC AAAAGCA
559-dNdCs4-Rv	5'-CACTAGTTATTACATAGCATCCGGACCACGACCAATACGC AAAAGCA
560-dNdCs4-Rv	5'-CACTAGTTATTACATAGCATCCGGACCACGACTAAGACGC ATAAGCA
561-dNdCs1-Fw	5'-TATGCTCGAGTCCCAACCTGTCCCCGAGATCGACACGCTG CGCGAAGAGATCGACCGG
562- dNR85s10-Rv	5'-CACTAGTTATTACATAGCATCCGGACCACGATCAAGACGC AAAAGCA
563-dNR85s10-Rv	5'-CACTAGTTATTACATAGCATCCGGACCACGACCAAGACGC AAAAGCAGGTTG
60-T7Pro	5'-TAATACGACTCACTATAGGG
